# Integrated transcriptomics and WGCNA reveal candidate hub genes associated with terpenoid biosynthesis in *Rehmannia glutinosa*

**DOI:** 10.3389/fpls.2026.1834043

**Published:** 2026-06-15

**Authors:** Linlin Xiao, Chengsi Lv, Haoyuan Li, Lijuan Yang, Xingyu Li, Qingxiang Yang, Peilei Chen, Jiuchang Su, Hongying Duan

**Affiliations:** 1 College of Life Sciences, Henan Normal University, Xinxiang, China; 2 Henan Engineering and Technology Research Center for Conservation and Utilization of Genuine Medicinal Herbs, Xinxiang, Henan, China; 3 Henan Biotechnology and Engineering Research Center of Green Medicinal Herbs, Xinxiang, Henan, China

**Keywords:** hub genes, *Rehmannia glutinosa*, terpenoid biosynthesis, transcriptomics, WGCNA

## Abstract

Terpenoids are the key bioactive constituents of *Rehmannia glutinosa*, a valuable medicinal plant. The rising economic and pharmaceutical values of *R. glutinosa* have necessitated elucidating the metabolic pathways governing terpenoid metabolism. Herein, we integrated transcriptome sequencing (RNA−seq) and weighted gene co−expression network analysis (WGCNA) to identify co−expression modules and hub genes closely linked to terpenoid biosynthesis in tuberous roots of cultivar ‘Wen85−5’ across eight developmental stages. A total of 20996 differentially expressed genes (DEGs) were identified, with GO/KEGG annotation analysis confirming enrichment in various metabolic and cellular processes. Further, we screened 16 terpenoid biosynthesis−related DEGs, mapping to the MVA (6 genes) and MEP (10 genes) pathways. WGCNA clustered 19957 DEGs into 16 modules, of which 9 modules (containing 28 hub genes) were potentially participated in the regulation of terpenoid biosynthesis. Functional annotation of these 9 modules revealed enrichment in secondary metabolic processes, as well as the biosynthetic pathways of terpenoids and polyketides, secondary metabolites, sesquiterpenes, and triterpenes. Among the 28 hub genes, 11 and 17 were mapped to the MVA and MEP pathways, respectively. Co-expression network analysis revealed intricate interactions between hub genes and between hub genes and key transcription factors. Notably, 11 of these hub genes exhibited conserved co-expression patterns across multiple modules and served as candidate genes potentially associated with terpenoid biosynthesis. The expression profiles of these 11 hub genes, inferred from FPKM values, were further validated by RT-qPCR, demonstrating consistent expression trends. This study provides the first systematic characterization of terpenoid biosynthetic network in *R. glutinosa*, offering critical insights and a valuable genetic resource for metabolic engineering to enhance terpenoid production.

## 1 Introduction

*Rehmannia glutinosa* Libosch., a perennial herb of the Scrophulariaceae family, is one of the most famous traditional Chinese herbs in China, possessing both pharmacological and economic value (Zhang et al., 2008a; Bian et al., 2023). *R. glutinosa* has garnered attention for its exceptional medicinal properties and holds a prominent position in traditional Chinese medicine (Zhang et al., 2008a; Bian et al., 2023). The tuberous roots and leaves of *R. glutinosa* are rich in various medicinal compounds, such as iridoid glycosides, glycosides, polysaccharides, amino acids, ionones, triterpenoids, phenylethanoid glycosides, and flavonoids (Dong et al., 2022; Chen et al., 2021), and these active components have positively pharmacological effects in the nervous, cardiovascular, cerebrovascular, and immune systems (Zhang et al., 2008b). Due to various biologically active ingredients, *R. glutinosa* has potential uses for anti-inflammatory (Zhang et al., 2023; Xie et al., 2024), anti-tumor (Lu et al., 2022), depression treatment (Wang et al., 2024), liver protection (Fu et al., 2020), and anti-aging activities (Wang et al., 2021a).

Among the numerous pharmacologically active compounds, terpenoids, a collective term for a class of natural compounds, are the most structurally and quantitatively abundant metabolites in plants, and are also rich in *R. glutinosa* (Zhang et al., 2013; Chen et al., 2021; Chen et al., 2022). More than 60000 terpenoids and their derivatives have been isolated and identified in nature (Brandt et al., 2009). Terpenoids play the important roles in maintaining normal growth and development, as well as resisting various adverse stresses in plants (Moses et al., 2013; Zeng et al., 2019). According to their functions, plant terpenoids can be divided into two categories: the primary and secondary metabolites (Bohlmann et al., 1998; Cheng et al., 2007; Tholl, 2015; Pichersky and Raguso, 2018; Yang et al., 2020). The primary metabolites mainly include plant hormones widely involved in plant growth and development (McGarvey and Croteau, 1995; Pichersky and Raguso, 2018), such as gibberellin, abscisic acid, and brassinolide, as well as carotenoids and chlorophyll involved in plant photosynthesis. Most terpenoids, including monoterpenes, sesquiterpenes, diterpenes, iridoid glycosides, etc (Bohlmann et al., 1998), in plants belong to secondary metabolites and play important roles in the interaction between plants and the external environment (Cheng et al., 2007; Tholl, 2015). According to reports, terpenoids are the key secondary metabolites and main active ingredients in *R. glutinosa*, in which over 70 types of terpenoid compounds were isolated and identified from *R. glutinosa* (Zhang et al., 2008a).

As shown in previous reports, terpenoids in higher plants are generated from the two independent pathways, the mevalonic acid (MVA) pathway in the cytosol and the methylerythritol 4-phosphate (MEP) pathway in plastids (Cheng et al., 2007). At present, research on terpenoids synthesis in plants mainly focuses on key enzymes of two pathways, as well as enzymes that catalyze terpenoid skeletons formation (Ma et al., 2021). There are two crossed components, isopentyl diphosphate (IPP) and its isomer dimethylallyl diphosphate (DMAPP), between the MVA and MEP pathways. IPP and DMAPP are the basic structural units that synthesized terpenoid biosynthesis (Wang et al., 2019). It has also been confirmed that the MEP pathway provides precursors IPP and DMAPP for the biosynthesis of terpenoids in *R. glutinosa* (Li et al., 2022). The farnesyl diphosphate (FPP) and geranylgeranyl diphosphate (GGPP) synthases belong to the isopentenyl transferase family and are the key enzymes of the terpenoid biosynthesis pathway in plants. They can catalyze IPP and DMAPP to form precursor substances of monoterpenes, sesquiterpenes, and diterpenes. Therefore, the FPP/GGPP synthase family is an important branch regulating terpenoid biosynthesis and plays an important role in the middle stage of terpenoid biosynthesis (Chen et al., 2021). GPP was synthesized by head-tail condensation of IPP and DMAPP (Tholl et al., 2004). Then, geraniol is generated from GPP under the catalytic action of geraniol synthase (GES) (Sung et al., 2011). Geraniol undergoes hydroxylation under the action of G10H to produce 10-hydroxygeraniol (Sung et al., 2011). Finally, 10HGO catalyzes the oxidation of 10-hydroxygeraniol at C1 and C10 positions to form a monoterpenoid compound, 10-oxygeranal (Verma et al., 2012). Starting from this step, the terpenoid biosynthesis was divided into two pathways (Peebles et al., 2011). Although some studies have reported the partly synthetic pathways of terpenoids, the potential pathway is still not fully understood in *R. glutinosa*. Therefore, the exploration of terpenoids’ synthetic pathways remains a huge challenge.

Weighted gene co-expression network analysis (WGCNA) facilitates the revelation of core gene networks based on the gene expression patterns from RNA-seq data (Wan et al., 2018; Guo et al., 2020), which is an effective method for network modeling based on simple networks and easily understandable statistical methods (Langfelder and Horvath, 2008), becoming a favored and popular technique in discovering hub factors controlling traits. WGCNA was developed for more effective analysis of datasets by combining high-throughput sequencing technology, which can quantify the correlation between genes and weigh the degree of correlation between gene pairs (Zhang and Horvath, 2005; Pei et al., 2017). Different from traditional research concentrating on a single gene or an isolated biomarker, WGCNA could investigate the co-expressed genes in the form of modules, and then the intramodular hub genes were extracted from co-expressed networks (Pei et al., 2017). In recent years, WGCNA has been widely used for various genomic studies to screen key genes in plants. WGCNA tools could divide genes with similar expression patterns into the same module, and then the correlation between these modules and specific characteristics or target traits was investigated to screen the hub genes highly associated with the specific characteristics or target traits (Meng et al., 2022; Xu et al., 2022; Azam et al., 2023; Yao et al., 2023; Gan et al., 2024; Yin et al., 2024). WGCNA also plays a crucial role in hub genes of compound synthesis (Pei et al., 2017). The hub genes involved in isoflavone accumulation in soybean seeds were identified by WGCNA (Azam et al., 2023). The hub genes related to oil content were revealed between the wild and cultivated soybean by WGCNA (Yao et al., 2023). Comparative transcriptomics and WGCNA uncovered the underlying mechanism of carotenoid synthesis (Lu et al., 2019). Due to the outstanding performance of WGCNA in revealing hub genes, we believe that it will help identify terpenoid synthetic pathways in *R. glutinosa*.

Herein, we obtained the transcriptome data related to the growth and development of *R. glutinosa* tubers from the NCBI public database. Afterwards, non-parametric transcriptome data analysis was conducted to explore hub genes associated with terpenoid biosynthesis during the growth and development process of *R. glutinosa* and the transcription factor (TF) families involved in regulating hub gene expression. Combined with WGCNA, further analysis was conducted to explore the interaction between hub genes and TFs. Finally, the gene expression of several hub genes associated with terpenoid biosynthesis was characterized by RT-qPCR. Our findings lay a theoretical foundation for future research on terpenoid biosynthesis and improving the accumulation of terpenoid metabolites in *R. glutinosa*.

## 2 Materials and methods

### 2.1 Raw RNA-Seq data acquisition

The raw data were acquired from the National Center for Biotechnology Information (NCBI) database (https://www.ncbi.nlm.nih.gov/) and downloaded by using the ‘prefetch’ command. By searching the keyword “*Rehmannia glutinosa*” in the NCBI SRA database, we obtained the RNA-Seq data. We found the original sequencing data of root growth and development of “Wen 85-5” *R. glutinosa* in the natural growth state. Then, all raw data were converted into standard FASTQ format files via the ‘FASTQ-dump’ command of the SRA Toolkit (v3.0.0) package.

According to the above filter criteria, the original sequencing data of 8 samples were selected for further analysis, and they were divided into the S groups (Accession: PRJNA221109) and Z groups (Accession: PRJNA197434) according to the development stage of *R. glutinosa*. Briefly, S group: the root samples were collected at three stages, including 30 (S1), 45 (S2), and 60 (S3) days after germination (Sun et al., 2015). The roots of five individual plants at three developmental phases were harvested and pooled together, and three independent cDNA libraries were prepared respectively from three developmental phases for RNA-Seq analysis. Z group: the collected roots were collected from five different stages (Li et al., 2015), including Z1 (7-8 cm of root length), Z2 (9-15 cm of root length), Z3 (1-2 cm of diameter column), Z4 (2-4 cm of diameter column), and Z5 (Maturity). The roots at five developmental stages were collected and pooled together, and six RNA-Seq libraries for three biological replicants of each sample were constructed.

### 2.2 Raw data quality control and assembly

To ensure that the raw reads are sufficiently high quality, low-quality reads were effectively removed to obtain clean reads by using Fastp (v0.23.2) (Ma et al., 2021). Then, we obtained basic information of raw reads, such as GC content, sequence length, and Q30 of each sample, from the quality inspection report of Fastp. Q30 represents the percentage of mass value of a certain base in total base number, in which the base proportion of Q30 should be higher than 85%. The clean data of each sample were filtered by Fastp, and were assembled and quantified to produce unigenes using Trinity (v2.4.0). Then, the clean reads were used for subsequent bioinformatic analysis. Since the raw data comes from different RNA-seq sequencing datasets, there may be batch effects between the raw data. To eliminate potential batch effects, the Combat-seq algorithm of sva package was applied, and corrected expression matrices were used for downstream differential expression and functional enrichment analyses.

### 2.3 Differentially expressed genes screening

Raw read counts of each gene were retained as the original input for differential expression analysis. Genes with extremely low expression were filtered out, retaining those with expression abundance above the threshold in at least 80% of all samples. The EdgeR package was used to identify DEGs from raw read counts (Robinson et al., 2010). The log_2_ FC (Fold Change), *p*-value, and False Discovery Rate (FDR) values of each unigene were calculated (Benjamini and Hochberg, 1995). In this study, genes with |log_2_FC|≥1 and FDR ≤ 0.05 were recognized as DEGs and selected for subsequent analysis. The FPKM (Fragments Per Kilobase of exon model per Million mapped fragments) values were widely used to evaluate and present the relative expression levels of unigenes (Mortazavi et al., 2008).

### 2.4 Functional annotation and enrichment analysis of DEGs

All DEGs or the genes from key modules were annotated to Gene Ontology (GO) and Kyoto Encyclopedia of Genes and Genomes (KEGG) database for functional annotation and pathway enrichment analysis. GO enrichment analysis was performed on the GO platform (https://www.geneontology.org/). The significantly enriched GO terms (*P* < 0.05) were further identified for the biological processes (BP), cellular component (CC), and molecular function (MF) (Meng et al., 2022). KEGG pathway enrichment analysis was conducted (http://www.genome.jp/kegg) (Yao et al., 2023). Terms with FDR<0.05 were considered significantly enriched. The bubble plots showing the top 20 terms or pathways were drawn by the OECloud online tool (https://cloud.oebiotech.com/).

### 2.5 Construction of weighted gene co-expression network analysis (WGCNA)

R software with the WGCNA package was used to create the WGCNA (Zhang and Horvath, 2005; Langfelder and Horvath, 2008). Firstly, the genes whose expression variance was too small were further filtered (Langfelder and Horvath, 2008), wherein the top 50% of genes with the highest variance across all samples were selected for WGCNA construction. The FPKM values of all genes and phenotype data of 8 samples were imported into WGCNA, and then correlation-based connections between phenotypes and gene modules were computed using the default settings. The networks were visualized by Cytoscape software (version 3.10.1; Ma et al., 2021). The gene expression matrix of transcriptome data was filtered, and then power values (β=12, soft threshold) were calculated and determined to select appropriate power values by the pickSoftThreshold function. Additionally, the mergeCutHeight parameter was 0.25, and different modules with a similarity of more than 75% were merged. We merged genes with similar expression patterns into the same module.

To better evaluate the correlation between gene expression patterns, the topological overlap matrix (TOM) was used to calculate the direct and indirect correlation between two genes, and then the TOM matrix was established (Lu et al., 2019). In this study, based on the dissimilarity of TOM values, the gene clustering and module division were carried out by the hierarchical average linkage clustering with the Dynamic Hybrid Tree Cut method (Langfelder et al., 2008; Zhan et al., 2015), which could group genes with similar patterns of expression. Afterwards, the genes were divided into different modules based on their expression pattern.

### 2.6 Identification and filtering of key modules

The identification of the key module was carried out according to a previous method (Yao et al., 2023; Gan et al., 2024). The correlation coefficients between Epigengene and different growth and development periods were calculated to quantify the degree of correlation between the module and different periods. A close relationship between the module and sample was indicated by a high expression of positive or negative eigenvalues in the module. Module-trait correlation heat maps were constructed to identify the modules that were correlated with the different growth and development periods. The modules with correlation coefficients≥| ± 0.65| and *P* < 0.05 were selected as key modules for further analysis.

### 2.7 Screening of hub genes in the specific module

The module membership (MM) values can be determined by analyzing the correlation between the gene expression and the corresponding module eigengene (Azam et al., 2023; Gan et al., 2024). The genes with a high |MM| value (top 10%) were selected in each module, and the gene is correlated with the module. The correlation analysis is performed between the gene expression and the corresponding phenotype value. The final value of the correlation coefficient is gene significance (GS; Azam et al., 2023). The hub genes are usually characterized by high GS (association between gene expression and traits) and MM (correlation between gene expression and module eigengene) values. Herein, the hub genes were screened by the criteria with MM ≥ 0.75 and GS ≥ 0.2 in each module.

### 2.8 Construction of a co-expression network of hub genes and/or TFs

All genes of key modules were used to construct a co-expression network by utilizing STRING (v12.0) (https://string-db.org/) (Szklarczyk et al., 2019). The encoding protein interactions between the hub genes and the other genes were predicted in the co-expression network. The interacted networks of hub genes and TFs were also constructed. The networks were visualized by Cytoscape v3.10.1 (Gan et al., 2024).

### 2.9 The real-time quantitative PCR (RT-qPCR) analysis of DEGs

The sequences of DEGs were obtained based on transcriptome sequencing results. The specific primers were designed by Primer Premier 5.0 (**Supplementary Table 1**). According to the instructions of the RNA extraction kit, the total RNA was extracted from the tissues of *R. glutinosa*. The first-strand cDNA was synthesized by HiScript II Q RT SuperMix for qPCR kit (Nanjing Vazyme Biotech Co., Ltd), following the experimental steps.

The RT-qPCR was conducted by Roche Light Cycler^®^96 automatic fluorescence quantitative PCR instrument (Roche, America) using ChamQ Universal SYBR qPCR Master Mix kit (Nanjing Vazyme Biotech Co., Ltd). *RgGADPH* and *RgTIP41* were used as an internal reference gene. Three technical replicates were set for each sample, and the relative expression levels of genes were calculated by 2^−ΔΔCT^ method (Livak and Schmittgen, 2001).

### 2.10 Statistical analysis

The experimental data were presented as the mean ± SD from three independent experiments, with triplicate for each. Statistical analysis was performed using SPSS 20.0 software with ANOVA. Bars with different letters indicated significant differences at a significance level of *P* < 0.05 based on Duncan’s test.

## 3 Results

### 3.1 The quality assessment of transcriptomic data and the analysis of differentially expressed genes

To investigate the differences in terpenoid levels of *R. glutinosa* at different growth and development periods, the transcriptomic data of 8 samples (S1-S3 and Z1-Z5) were collected. After filtering and quality controlling, 52514582 clean reads and 25729071870 bp clean bases were obtained (**Supplementary Table 2**). The percentage of Q30 bases in each sample was 90.73-96.36%, and GC content was 42.83-47.33%. The 42843 transcripts and 39915 unigenes were obtained by Trinity software. A series of parameters (**Supplementary Table 2** and **S3**) indicated that the raw sequencing data were of high quality and could be used for subsequent analysis.

**Figure 1:**
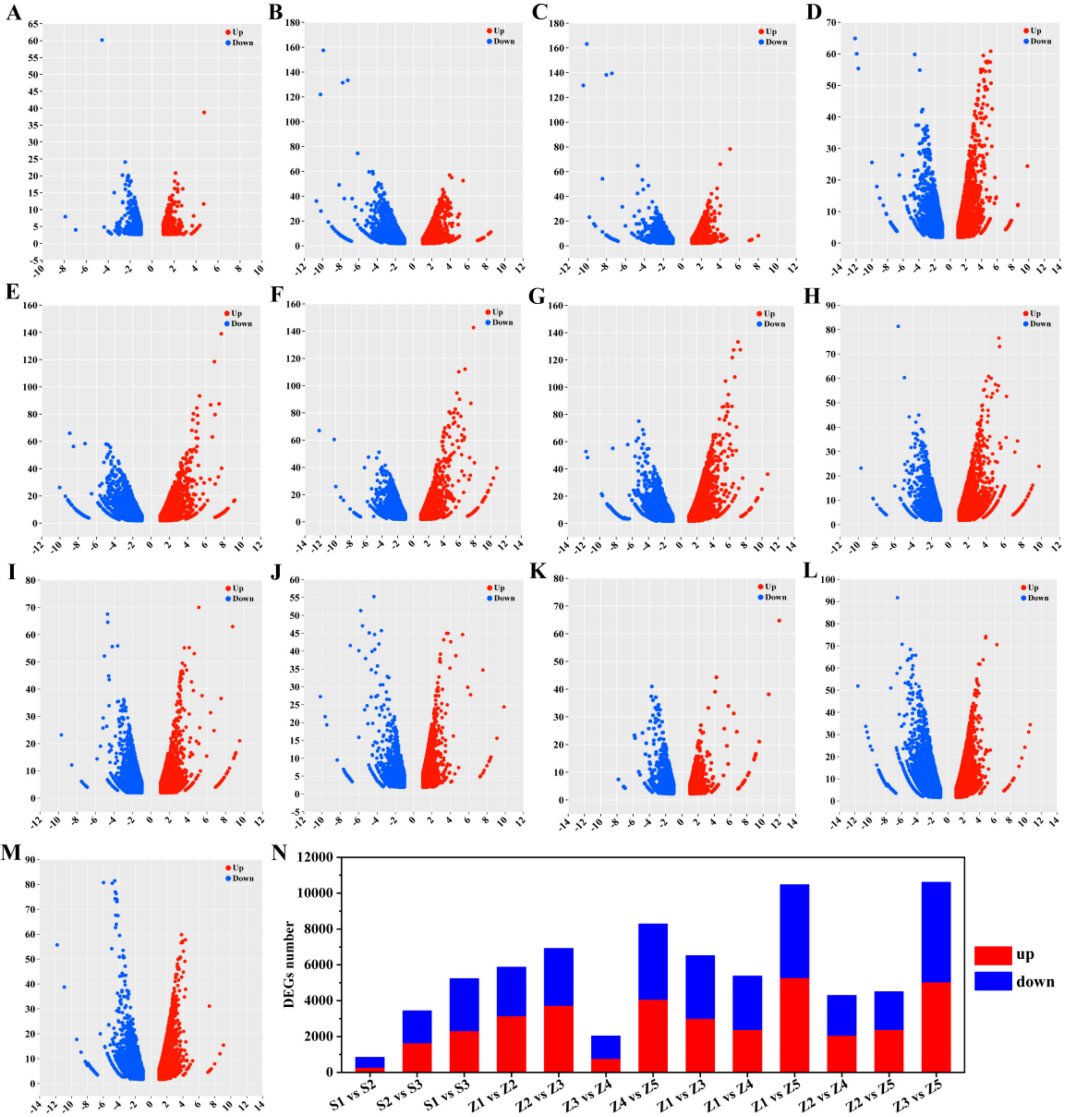
Analyses of differentially expressed genes (DEGs). **(A–M)** The volcanic plot of DEGs of each comparison. Red dots indicated the upregulated DEGs (up). Blue dots represented the downregulated DEGs (down). **(N)** Number of DEGs in all comparison groups.

After pairwise differential expression analysis using raw read counts via EdgeR, all DEGs from different comparison groups were merged and deduplicated. Finally, a total of 20996 DEGs were identified across all comparisons, including 6285 of S group and 14711 of Z group. In addition, we also analyzed the number of DEGs between two different groups in S Group and Z Group, respectively. 827, 3430, and 5221 DEGs were identified, respectively, between S1 and S2, S2 and S3, and S1 and S3 (**Figures 1A–C**). The number of DEGs in Z1 vs Z2, Z1 vs Z3, Z1 vs Z4, and Z1 vs Z5 (**Figures 1D–G**) was 5865, 6506, 5365, and 10467, respectively. 6907, 4283, and 4491 DEGs were identified, respectively, in Z2 vs Z3, Z2 vs Z4, and Z2 vs Z5 (**Figures 1H–J**). A total of 2021, 10606, and 8274 DEGs were identified, respectively, between Z3 and Z4, Z3 and Z5, and Z4 and Z5 (**Figures 1K–M**). The number of DEGs between different groups was also listed (**Figures 1N**).

### 3.2 GO and KEGG functional annotation of DEGs

**Figure 2:**
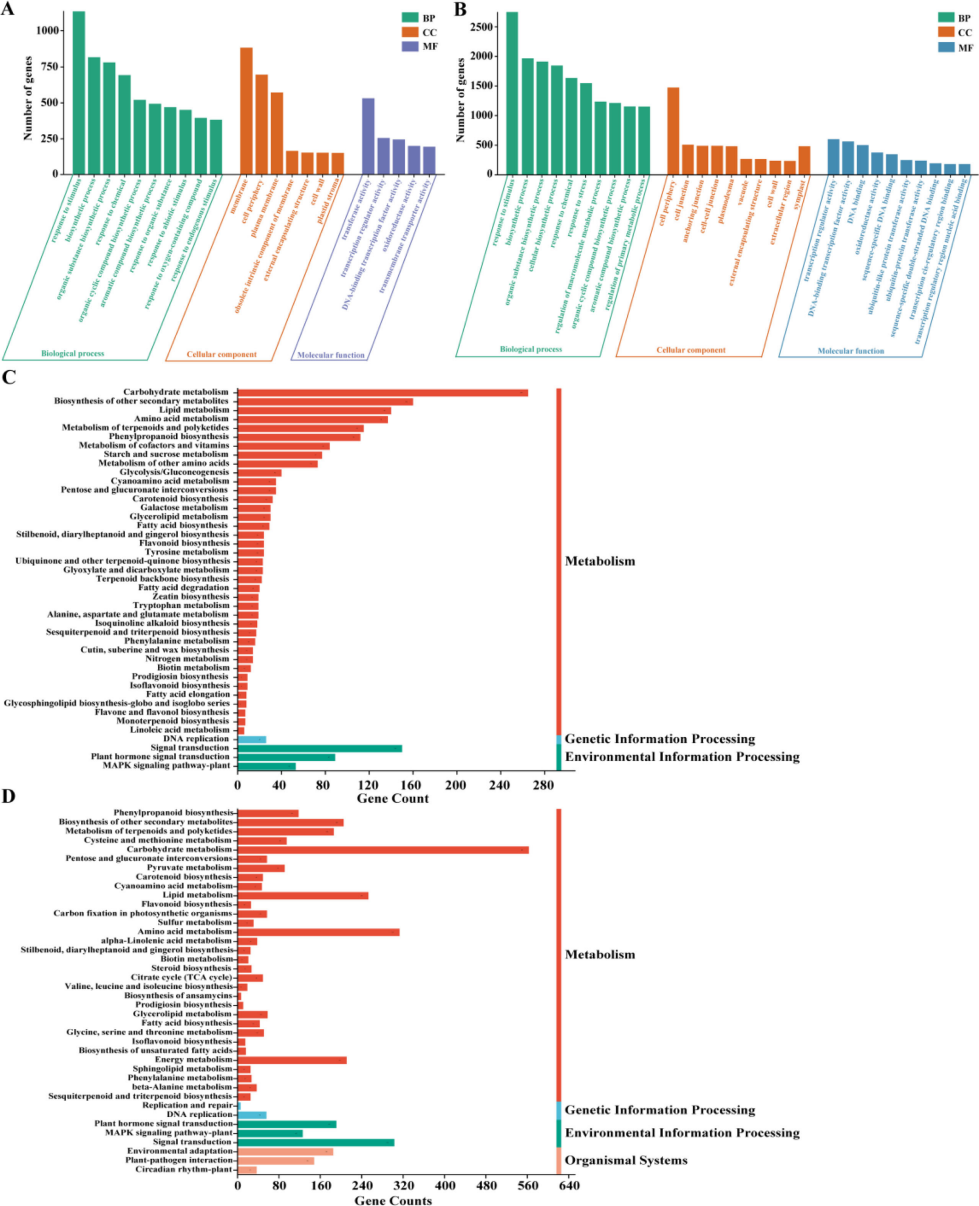
GO and KEGG annotation classification of DEGs. GO annotation of DEGs in S **(A)** and Z **(B)** groups. GO enrichment analysis of the DEGs highlighted the key biological processes, cellular components, and molecular functions. KEGG annotation of DEGs in S **(C)** and Z **(D)** groups. KEGG pathway enrichment analysis of the DEGs showed the significant pathways associated with the various metabolic processes.

GO analysis of DEGs from S group was shown in **Figure 2A**. In BP, 1132, 814, and 778 DEGs were mainly enriched in response to stimuli and the organic matter biosynthesis process. In CC, DEGs were mainly enriched in the membrane, cell periphery, and plasma membrane, with 880, 693, and 569 DEGs, respectively. In MF, 529, 253, and 243 DEGs were mainly enriched in transferase activity, transcriptional regulatory activity, and DNA-binding transcription factor activity. GO analysis of DEGs from Z group was shown in **Figure 2B**. In BP, 2759, and 1966 DEGs were mainly enriched in response to stimuli and the organic matter biosynthesis process, respectively. In CC, DEGs were mainly enriched in the cell periphery, cell junctions, and anchored junctions, with 1475, 508, and 489 genes, respectively. In MF, 601 and 564 DEGs were mainly enriched in transcriptional regulatory activity and DNA binding transcription factor activity, respectively. In summary, the abundant DEGs of S and Z groups were participated in metabolic processes.

KEGG annotation of DEGs from S and Z groups was conducted to explore their function at different developmental stages. In 43 pathways of S group, DEGs were mainly enriched in carbohydrate metabolism and biosynthesis of other secondary metabolites, accounting for 4.21% and 2.54%, respectively (**Figure 2C**). In 40 pathways of Z group, DEGs were mainly annotated in carbohydrate metabolism and biosynthesis of other secondary metabolites, accounting for 3.83%, and 1.39%, respectively (**Figure 2D**). In summary, the abundant DEGs of S and Z groups were participated in secondary metabolites biosynthesis.

### 3.3 The expression analysis of key genes related to terpenoid biosynthesis

**Figure 3:**
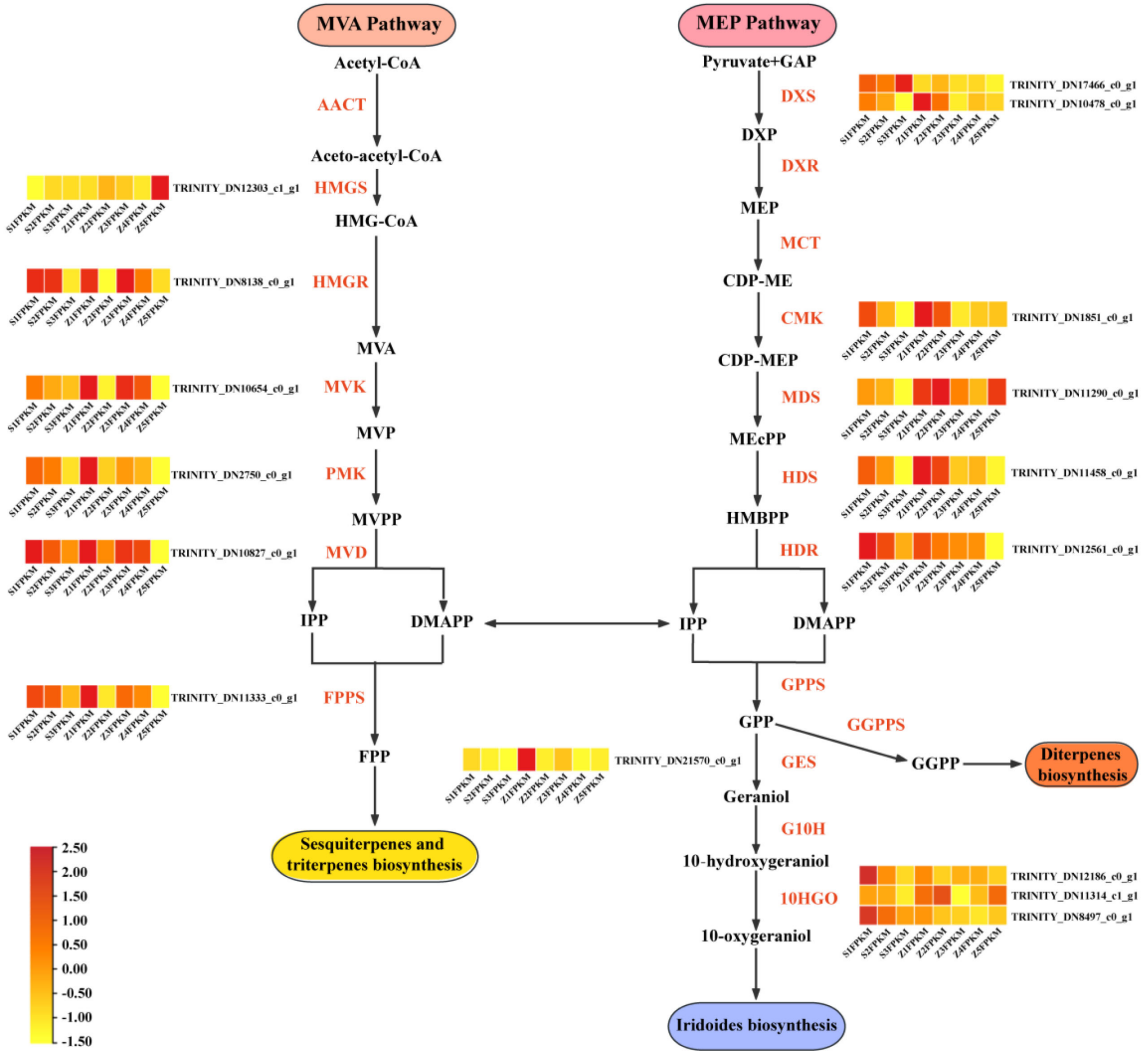
Heat map of DEGs expression in the terpenoid synthesis pathway in *R. glutinosa* at 8 stages according to the transcriptome database. The differential expressions of DEGs in the biosynthetic pathway of terpenoid compounds, including sesquiterpenes, triterpenes, diterpenes, and iridoides biosynthesis. Note: Yellow and red indicated, respectively, the relative expression levels of genes from low to high.

16 DEGs related to terpenoid biosynthesis were identified (**Supplementary Table 4** and **S5**). To compare the expression of DEGs at different developmental stages, their expression levels were shown based on FPKM values (**Figure 3**). 1 *RgHMGS*, 2 *RgDXS*, 1 *RgCMK*, 1 *RgHDS*, and 1 *RgHDR* were involved in terpenoid skeleton synthesis. Additionally, *RgHMGR*, *RgMVK*, *RgPMK*, *RgMVD*, and *RgFPPS* were related to sesquiterpenes and triterpenoids biosynthesis, in which 5 DEGs were highly expressed during S1, Z1, Z3, and Z4. *RgFPPS* showed high expression during Z3 and Z4, indicating that sesquiterpenes and triterpenoids may have begun to accumulate from Z3. 2 *RgDXS*, 1 *RgCMK*, 1 *RgMDS*, 1 *RgHDR*, 1 *RgCES*, and 3 *Rg10HGO* were involved in iridoid glycosides biosynthesis, of which 6 DEGs were highly expressed during Z1 and Z2. The expression of 3 *Rg10HGO* showed a trend of first decreasing and then increasing after Z1, indicating that iridoid glycosides accumulation was higher in the swelling and maturation stage.

### 3.4 WGCNA analysis and key modules identification

After TPM standardization, there was a total of 39915 genes in the gene expression profile. Then, the approximately top 50% of genes with the highest variance were screened using the R program. The expression data of 19957 genes were obtained as the input dataset for WGCNA. All samples exhibited high similarity (**Supplementary Figure 1A**), and no outlier samples were found. Therefore, all samples were suitable for subsequent analysis. To excavate co-expression modules and construct a network, a soft threshold power (β=12) was calculated by pickSoftThreshold, and when R²>0.85, the average connectivity tends towards 0 (**Supplementary Figures 1B, C**).

**Figure 4:**
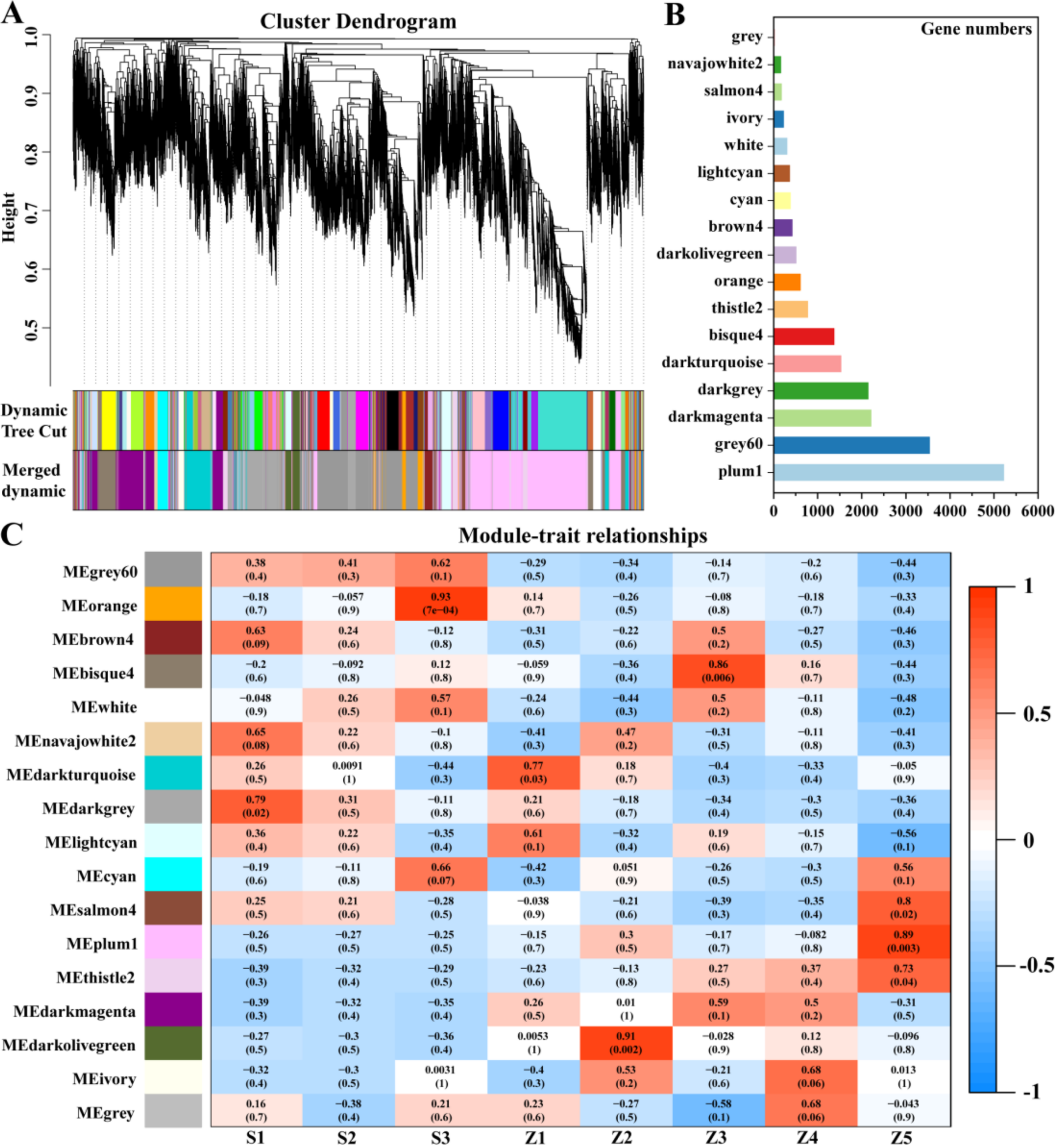
Gene clustering tree construction and modules identification, gene numbers in each module, and heat map of correlations between modules and traits. **(A)** Clustering dendrograms of genes and module division were obtained by clustering the dissimilarity. Each module was indicated by colors in the row, which contained a group of highly connected genes. **(B)** Distribution of gene number in modules. The vertical axis represented different modules, and the horizontal axis represented gene numbers. **(C)** The correlation relationship between the module and the stage of growth and development. Each row in the table represented a module (left), and each column represented a development stage (below). The table was colored according to the correlation with a legend on the right. Note: Red and blue grids represented a positive and negative correlation, respectively. The values inside and outside the parentheses are p-values and correlation coefficient (r), respectively.

Next, a gene clustering tree was constructed based on the correlation of gene expression, in which each branch corresponds to a set of genes with highly correlated expression (**Figure 4A**). To integrate modules with similar expressions, a gene co-expression network was constructed by the Dynamic Hybrid Tree Cut method. Finally, all genes were divided into 16 modules with different colors (**Figure 4A**). Due to exhibiting a scattered distribution characteristic, the genes within the gray module showed no significant co-expression trends, and thus, it was not further analyzed. Gene numbers in each module were shown in **Figure 4B**. Among them, Plum1 module had the most genes (5220), and Navajowhite2 module had the fewest genes (162).

Modular eigenvalues (ME) and their correlation with traits were calculated to identify modules that were significantly associated with growth and development traits. Here, 6 specific modules related to growth and development were identified (**Figure 4C**). Darkgrey, Orange, and Darkturquoise were potentially participated in the regulation of root elongation (S1), root maturation (S3), and root elongation (Z1), respectively. Darkolivegreen, Bisque4, and Plum1 were closely associated with root enlargement (Z2), metaphase of root enlargement (Z3), and anaphase of root enlargement (Z5), respectively. 9 potentially correlated modules were identified, including Grey60, Lightcyan, Darkgrey, Orange, Darkmagenta, Plum1, Darkturquoise, Thistle2, and Salmon4 (**Supplementary Table 6**).

### 3.5 GO and KEGG analysis of DEGs related to terpenoid biosynthesis in WGCNA

**Figure 5:**
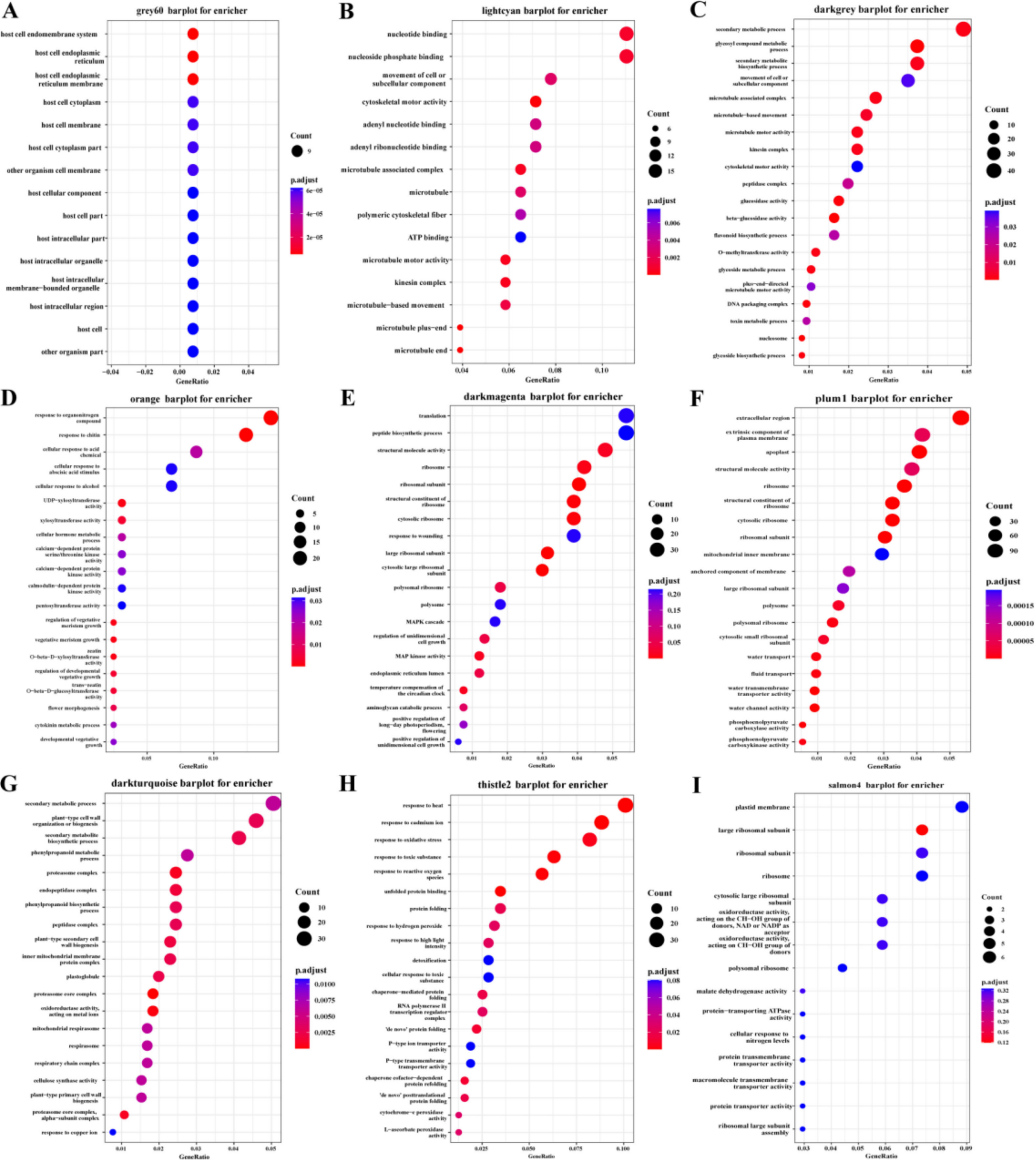
GO functional enrichment of co-expression modules related to terpenoid synthesis **(A–I)**. The horizontal axis represented enrichment factors, the vertical axis showed metabolic pathway names, the color of the dots is p-value value, red indicated significant enrichment, and the size of the dots represented the number of enriched DEGs.

To further explore the gene function in each module, all genes within the 9 modules were screened by GO analysis, including BP, CC, and MF (**Figure 5**; **Supplementary Table 7**). Darkrey and Darkturquoise were mainly enriched in secondary metabolic processes and secondary metabolite biosynthetic processes. Grey60 was evenly enriched in 15 CC. Lightcyan was mainly concentrated in nucleotide binding and nucleoside phosphate binding. Orange was mainly enriched in response to organonitrogen compounds and chitin. Darkmagenta mainly concentrated on transcription, peptide biosynthesis, and structural molecular activity. Plum1 and Salmon4 were mainly enriched in the extracellular region and plastid membranes, respectively. Thistle2 was mainly enriched in response to heat, cadmium ions, and oxidative stress. GO analysis showed that the key genes of Darkrey and Darkturquoise were mainly enriched in secondary metabolism and secondary metabolite biosynthesis.

**Figure 6:**
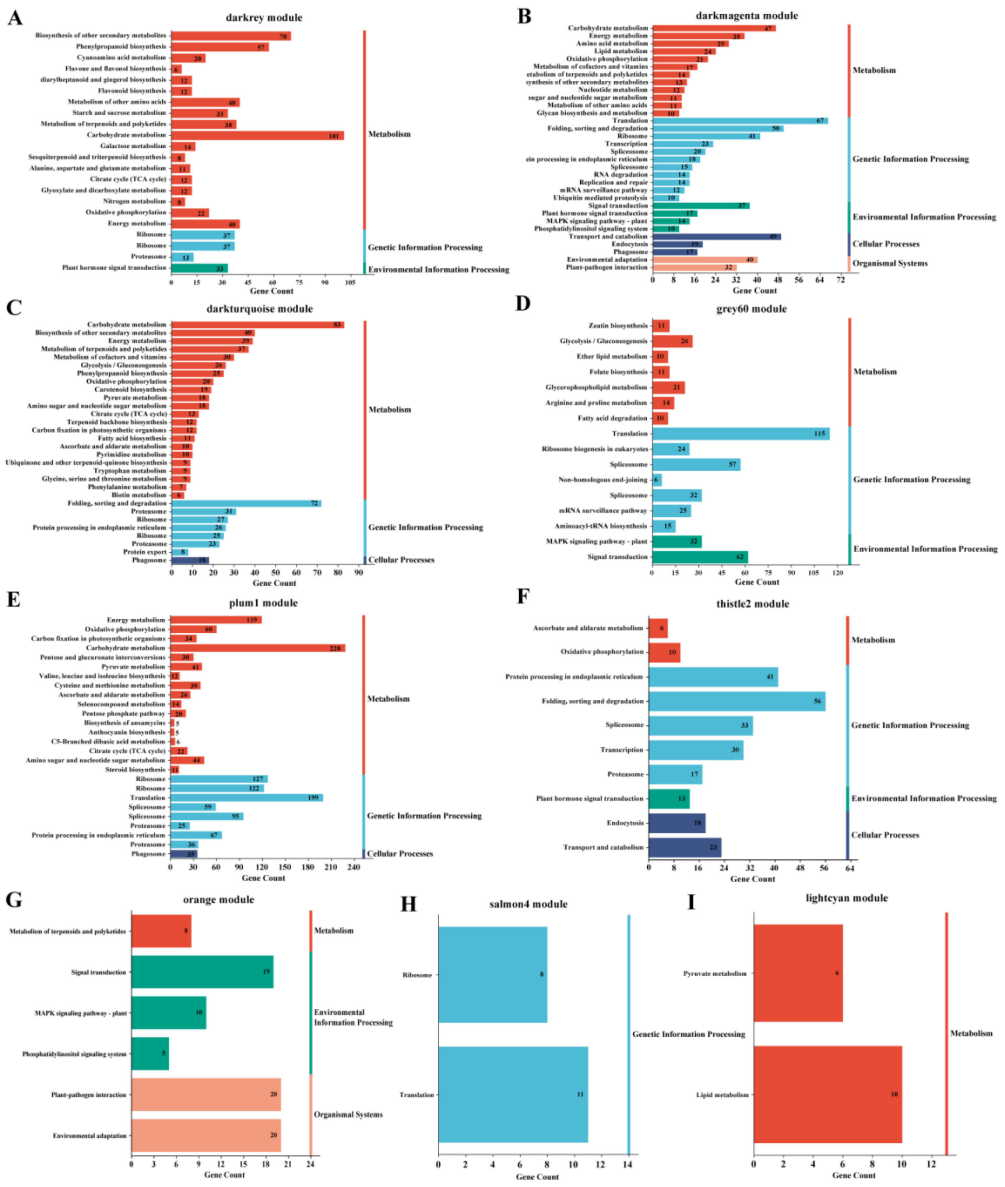
KEGG enrichment analysis of hub genes related to terpenoid synthesis in co-expressed modules **(A–I)**. The horizontal axis represented the gene count of KEGG terms. The vertical axis indicated KEGG enrichment terms.

Next, 9 modules were screened by KEGG analysis (**Figure 6**). Darkrey, Darkmagenta, or Darkturquoise mainly concentrated on the metabolism of terpenoids and polyketides, biosynthesis of other secondary metabolites, or biosynthesis of sesquiterpenes and triterpenes. Grey60 and Plum1 were mainly enriched in transcription, glycolysis/gluconeogenesis, or carbohydrate metabolism. Thistle2, Orange, Salmon4, and Lightcyan were mainly concentrated in oxidative phosphorylation, metabolism of terpenoids and polyketides, or lipid metabolism. KEGG analysis showed that, except Grey60 and Plum1, other modules were mainly enriched in biosynthesis of terpenoids and polyketides, other secondary metabolites, or sesquiterpenes and triterpenes.

### 3.6 Expression analysis of key genes in each module related to terpenoid biosynthesis

**Figure 7:**
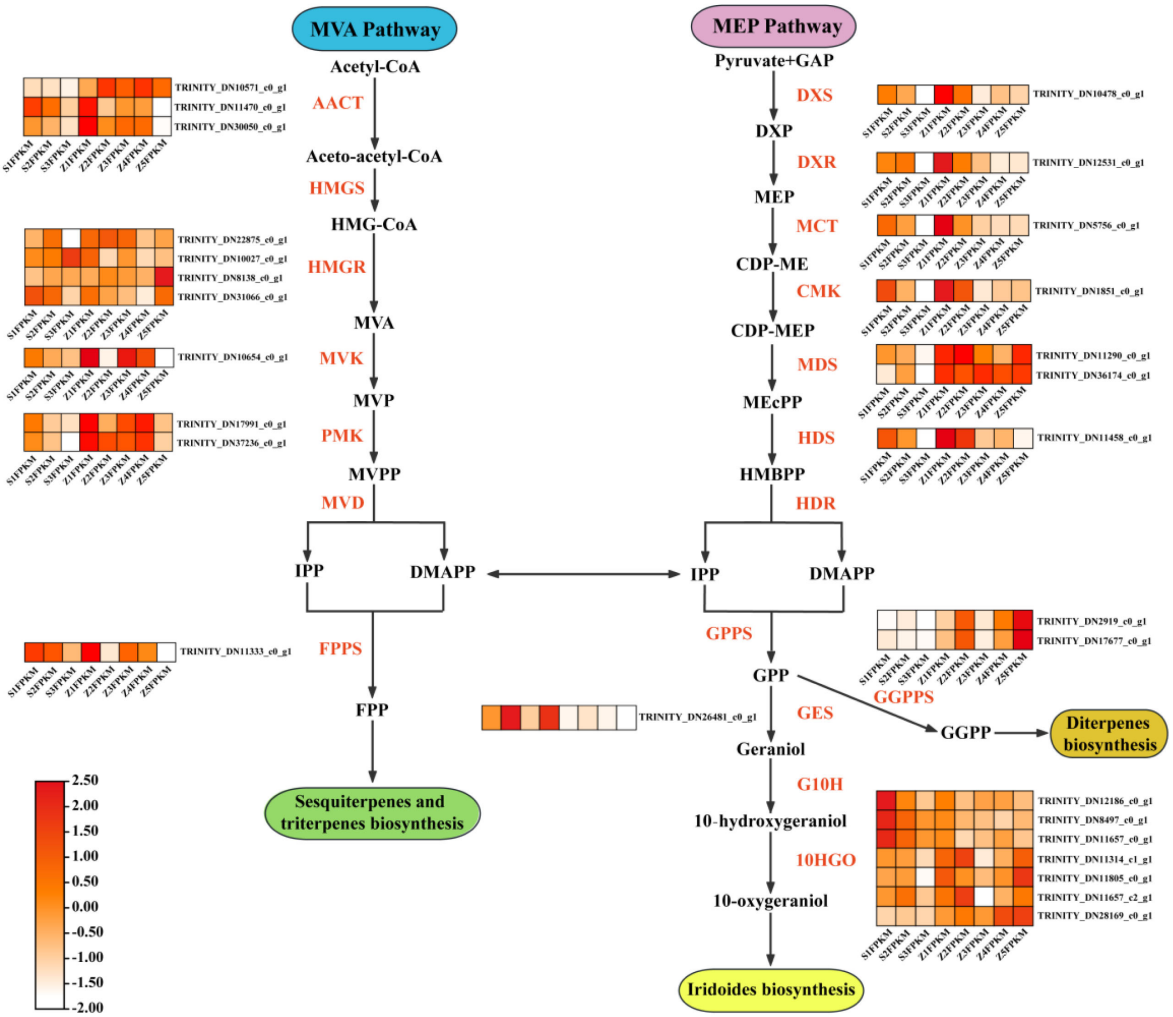
The expression heatmap of co-expressed genes related to terpenoid skeleton biosynthesis of the different modules from WGCNA. The differential expressions of the hub genes were shown in MVA and MEP pathways of terpenoid compounds, including sesquiterpenes, triterpenes, diterpenes, and iridoide biosynthesis. Note: Light white and dark red indicated, respectively, the relative expression levels of genes from low to high.

Further, FPKM values of 28 hub genes (co-expressed genes related to terpene skeleton biosynthesis in different modules of WGCNA, **Supplementary Table 6**) were used to demonstrate the gene expression during at different developmental stages (**Figure 7**). Among them, 10 genes may encode 6 enzymes in MVA pathway, including 3 *RgAACT*s (*RgAACT1* of Grey60, *RgAACT2* of Lightcyan, and *RgAACT3* of Darkmagenta), 4 *RgHMGR*s (*RgHMGR1* of Grey60, *RgHMGR2* of Orange, *RgHMGR3* of Plum1, *RgHMGR4* of Salmon4), *RgMVK1* (Darkmagenta), and 2 *RgPMK*s (*RgPMK1* and *RgPMK2* of Darkmagenta). In MEP pathway, *RgDXS1*, *RgDXR1*, *RgMCT1*, *RgCMK1*, *RgHDS1* were found in Darkturquoise, and *RgMDS1* and *RgMDS2* came from respectively Plum1 and Thistle2. Plum1 contains *RgFPPS1*, which might be involved in sesquiterpenes biosynthesis. Plum1 contained *RgGGPS1* and *RgGGPPS2*, which might be involved in diterpenes biosynthesis. Plum1 also contained *RgGES1*, *Rg10HGO4*, *Rg10HGO5*, and *Rg10HGO6*. At the branch end of iridoides biosynthesis pathway, there are *Rg10HGO1*, *Rg10HGO2*, and *Rg10HGO3* in Darkgrey. There was *Rg10HGO*7 in Thistle2.

### 3.7 Construction of co-expression network of DEGs and TFs involved in terpenoid biosynthesis

**Figure 8:**
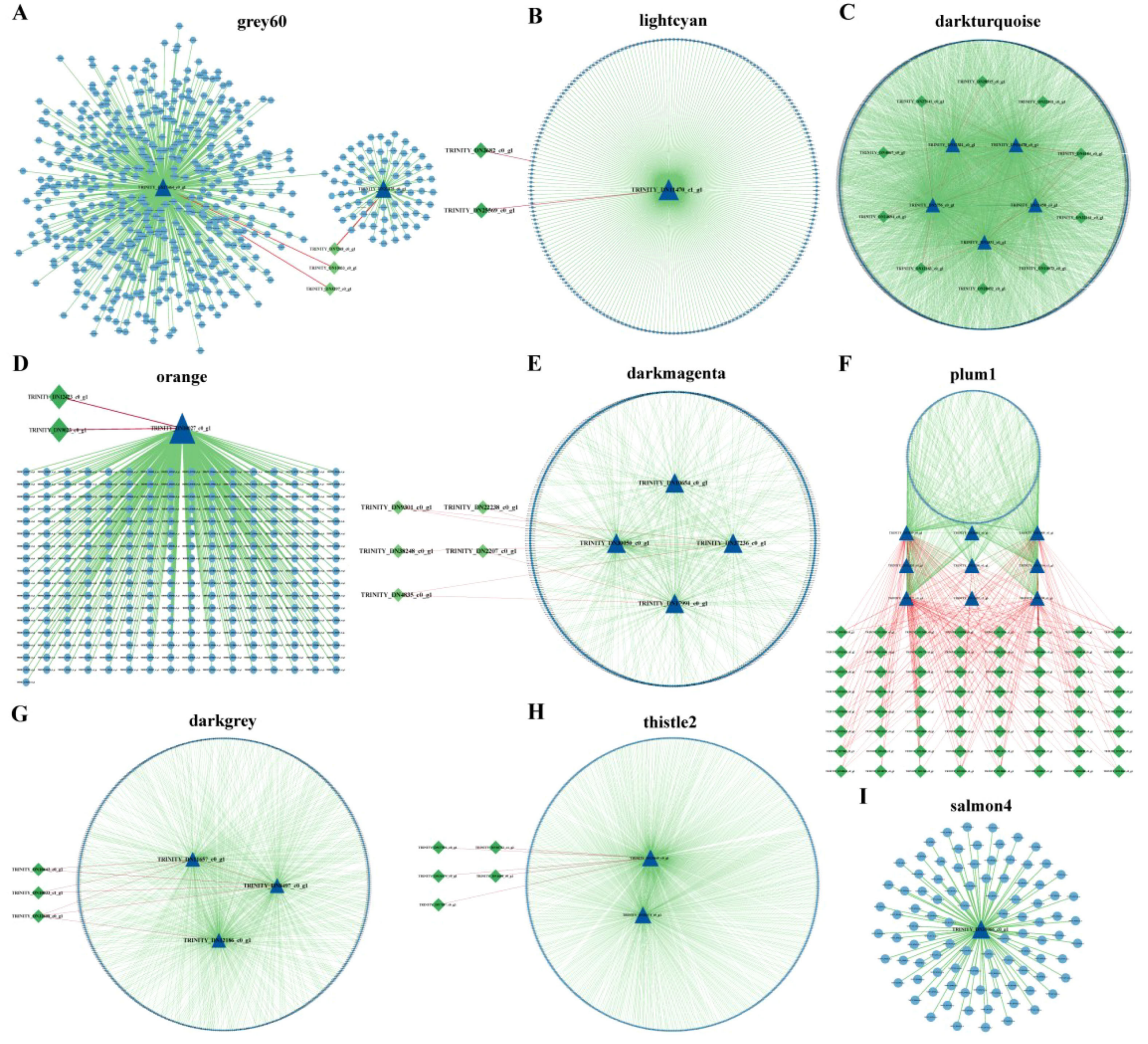
Co-expression network of hub genes related to terpenoid synthesis in 9 modules **(A–I)**. Regulation networks of hub genes with 9 modules were constructed respectively. In the network of co-expression module, blue triangles represent one gene related to terpenoid synthesis, blue circles represent an interacting gene, and one green diamond represents one transcription factor.

Co-expression networks of 28 hub genes and TFs were constructed, respectively, to investigate the relationships among genes, as well as the crosstalk between genes and TFs. TFs regulating terpenoid biosynthesis in 9 modules were listed in **Supplementary Table 8**. In the co-expression network of Grey60, *RgACT1* and *RgHMGR1*, no co-expression relationship was observed, and they were divided into 2 independent parts (**Figure 8A**), in which *C3H*, *HD-ZIP*, or Trihelix regulated *RgACT1* or *RgHMGR1* expression. In Lightcyan, *HSF* and *NAC* regulated *RgACT2* expression (**Figure 8B**). In Darkturquoise, *RgDXS1*, *RgDXR1*, *RgMCT1*, *RgCMK1*, and *RgHDS1* showed the co-expression relationships (**Figure 8C**), of which 10 TFs regulated the expression of five genes. In Orange, *GRAS* and *WRKY* regulated *RgHMGR2* expression (**Figure 8D**). In Darkmagenta, 4 hub genes, there is no direct interaction, exhibiting co-expression relationships (**Figure 8E**), where *MYB-related*, *C3H*, *GeBP*, *GRAS*, and *NF-YC* regulated *RgACT3*, *RgPMK1*, or *RgPMK2* expression. However, no TFs regulated *RgMVK1* were found. In Plum1, the interaction among *RgMDS1*, *RgGGPPS1*, and *RgGGPPS2*, the interaction among *RgFPPS1*, *RgGGPPS2*, and *Rg10HGO6*, and the interaction between *Rg10HGO4* and *Rg10HGO6* were showed (**Figure 8F**). In Plum1, 64 TFs were identified, of which *bHLH*, *ERF*, and *GRAS* have the highest proportion (12.5%). In Darkgrey, *Rg10HGO2* and *Rg10HGO3* showed the interaction (**Figure 8G**), while *MYB-related*, *HD-ZIP*, and *WRKY* regulated *Rg10HGO2* and *Rg10HGO3* expression. In Darkgrey, *Rg10HGO1* exhibited no significant interaction with the other two genes; however, it is closely related to *MYB*. In Thistle2, *RgMDS2*, and *Rg10HGO7*, they do not interact, exhibiting a co-expression relationship (**Figure 8H**), in which, except for *RgMDS2*, all five TFs regulated *Rg10HGO7* expression. In Salmon4, no TFs regulated *RgHMGR4* expression (**Figure 8I**).

### 3.8 Expression levels of key genes by FPKM values and RT-qPCR analysis

**Figure 9:**
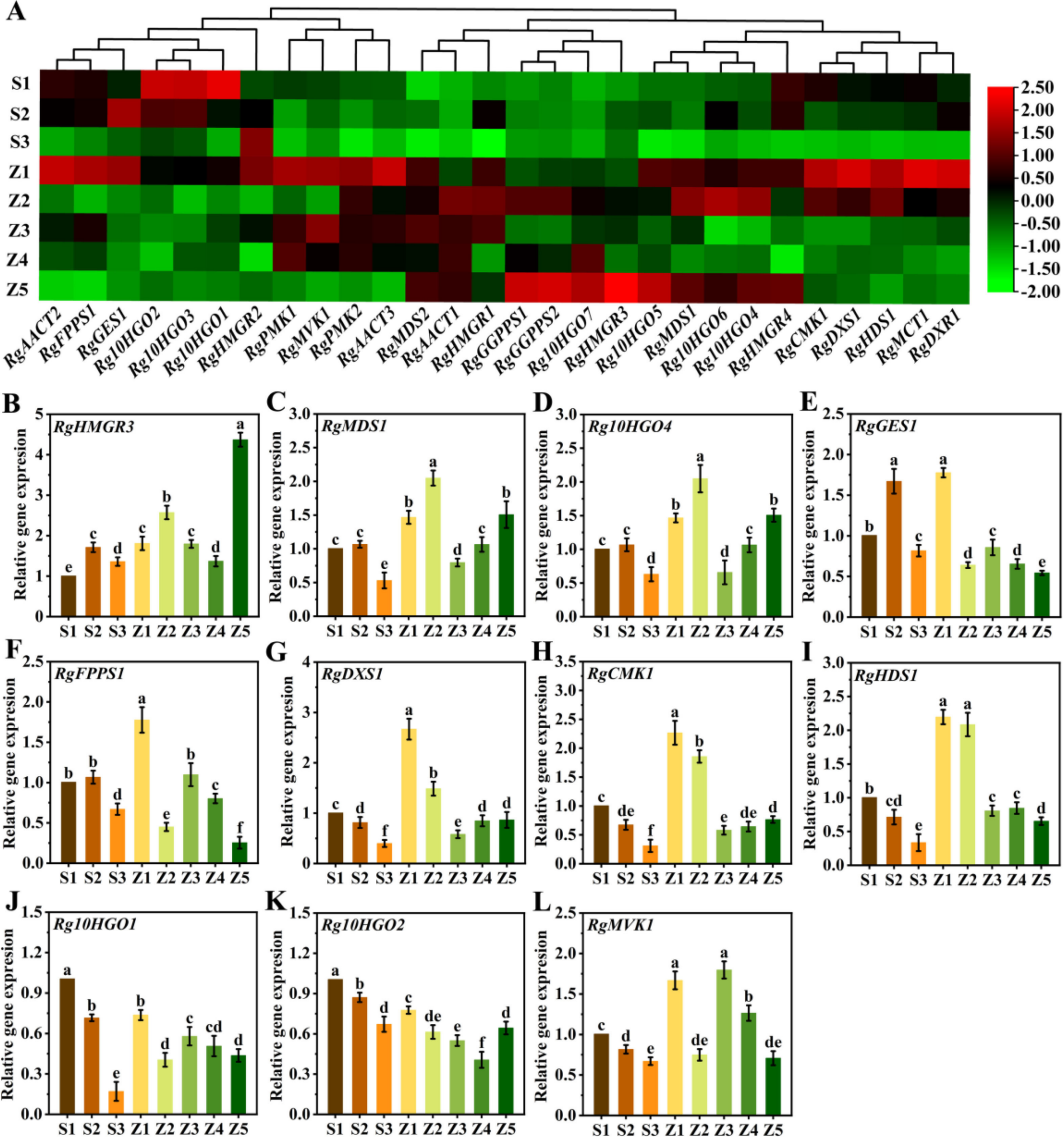
Expression levels of key genes involved in terpenoid biosynthesis by FPRM and RT-qPCR verification. **(A)** Heat map of key gene expression involved in terpenoid biosynthesis by FPRM values. Note: Dark green and dark red indicate the relative expression levels of genes from low to high. **(B–L)** Expression levels of key genes involved in terpenoid biosynthesis by RT-qPCR. All data were from three biological replicates (mean ± SD). Bar with the letters indicated significant differences, as determined by a one-way analysis of variance (ANOVA) with Duncan’s multiple range tests at *P* < 0.05 levels.

To verify the roles of hub genes in terpenoid biosynthesis of *R. glutinosa*, the expressed differences of 28 genes were first compared by their FPKM values during S1-S3 and Z1-Z5 (**Figure 9A**). Among the 28 genes, 11 genes, including *RgHMGR3*, *RgFPPS1*, *RgMDS1*, *RgGES1*, *Rg10HGO4*, *RgMVK1*, *RgDXS1*, *RgCMK1*, *RgHDS1*, *Rg10HGO1*, and *Rg10HGO2*, were not only co-expressed in corresponding module, but also hub genes for terpenoid biosynthesis. In Plum1 related to Z5, the expression levels of *RgHMGR3*, *RgMDS1*, and *Rg10HGO4* were higher, while the expression levels of *RgGES1* and *RgFPPS1* were relatively lower. In Darkmagenta, *RgMVK1* expression was relatively high during Z1 and Z3, showing a trend of first decreasing and then increasing. In Darkturquoise related to Z1, the expression levels of *RgDXS1*, *RgCMK1*, and *RgHDS1* were higher. In Darkgray related to S1, *Rg10HGO1* and *Rg10HGO2* expressions were higher. We speculated that 11 hub genes play the crucial roles in regulating terpenoid biosynthesis during different stages of growth and development in *R. glutinosa*.

Next, the expression levels of 11 hub genes were checked by RT-qPCR (**Figures 9B–L**). As expected, *RgHMGR3* expression was the highest in Z5, suggesting that RgHMGR3 played an important role in maturity stage. The expression levels of *RgMDS1* and *Rg10HGO4* were the highest in Z2. *RgHDS1* expression was the highest during both Z1 and Z2. We speculated that RgMDS1, Rg10HGO4, and RgHDS1 were the main players in Z2. The highest expression of *RgFPPS1*, *RgDXS1*, and *RgCMK1* was observed in Z1. The transcript level of *RgGES1* was the highest during both S2 and Z1. *RgMVK1* expression was the highest during both Z1 and Z3. These findings indicated that RgHDS1, RgFPPS1, RgDXS1, RgCMK1, RgGES1, and RgMVK1 exerted the main catalytic function in Z1. RgGES1 and RgMVK1 were the key enzymes, respectively, in S2 and Z3. Additionally, the highest expression levels of *Rg10HGO1* and *Rg10HGO2* were found in S1, implying that Rg10HGO1 and Rg10HGO2 were the critical players in S1.

## 4 Discussion

*R. glutinosa* is a perennial herbaceous plant in the Scrophulariaceae family, commonly used as medicine for its tuberous roots, which are one of the traditional medicinal materials (Zhang et al., 2008a; Bian et al., 2023). Its active components are mainly terpenoids, which have a wide range of pharmacological activities (Zhang et al., 2008a; Chen et al., 2021; Zhang et al., 2013). However, the regulatory mechanisms underlying terpenoid metabolism in *R. glutinosa* remain incompletely elucidated, making it crucial to investigate the key genes and metabolic networks involved in terpenoid biosynthesis. Herein, RNA-seq data retrieved from NCBI database were utilized to analyze DEGs during the growth and development stages of *R. glutinosa*. DEGs related to terpenoid biosynthesis were screened, and then a co-expression network was constructed through WGCNA to explore the crosstalk between DEGs and potential TFs.

Herein, Trinity assembly generated 42843 transcripts and 39915 unigenes. Although the reference genome of *Rehmannia chingii* is available, moderate genetic divergence exists between *R. glutinosa* (tetraploid) and *R. chingii* (diploid). Using a heterologous reference genome would cause low read mapping efficiency, misalignment, and loss of species-specific transcripts. To comprehensively capture stage-specific unique transcripts, and ensure the accuracy of subsequent bioinformatics analysis, we adopted Trinity *de novo* assembly. A total of 8 transcriptome data (S1-S3 and Z1-Z5) covering early and full developmental stages of *R. glutinosa* were used to screen DEGs, after screening and quality control (**Supplementary Table 2**). We acknowledged that 8 samples used for WGCNA were relatively smaller than the conventional recommended minimum, which might bring potential interference to module division and hub gene identification. Although we adopted rigorous parameter optimization and quality control, co-expression modules and hub genes still need further validation with an expanded sample and experiments. DEGs among 8 groups were shown in **Figure 1**. GO and KEGG enrichment showed that these DEGs of S and Z groups were significantly enriched in secondary metabolic processes, especially terpenoid biosynthesis (**Figure 2**). Terpenoid biosynthesis in plants relies on two parallel and spatially separated pathways: MVA pathway and MEP pathway. In this study, multiple rate-limiting genes in both pathways were identified as DEGs, including *RgHMGR*, *RgAACT*, *RgMVK*, *RgPMK* in MVA pathway, and *RgDXS*, *RgDXR*, *RgMCT*, *RgCMK*, *RgHDS*, *RgMDS* in MEP pathway (**Supplementary Table 5**; **Figure 3**). These genes showed distinct but coordinated expression patterns across developmental stages, indicating their non−redundant roles in controlling biosynthesis of terpenoid precursors. Our results showed that MEP pathway genes were highly activated at early stages, suggesting a supply of terpenoid skeleton construction. In contrast, MVA pathway genes maintained relatively stable expression and were gradually enhanced along with root development, supporting continuous terpenoid production. These ensured efficient precursor supply, thus contributing to terpenoids accumulation. Downstream genes, including *RgFPPS*, *RgGGPPS*, *RgGES*, and *Rg10HGO*, were also differentially expressed (**Figure 3**). The high expression of these genes at middle and late stages indicated that terpenoid accumulation was mainly enhanced during tuberous root expansion and maturation, which was consistent with the pattern that the secondary metabolites accumulated largely in middle and late stages (Tan et al., 2008; Xu et al., 2014; Chen et al., 2021).

WGCNA is a systems biology approach based on gene expression data for discovering gene co-expression networks and functional modules. Related studies have shown that the WGCNA method provides a new approach for screening key candidate genes and co-expression modules related to plant growth and development, as well as the accumulation of secondary metabolites (Hollender et al., 2014; Bai et al., 2015; Garg et al., 2017; Mellidou et al., 2021). In recent years, the application of WGCNA has shown a rapid development trend. A co-expression network for DEGs related to the growth and development of tuberous roots was constructed by WGCNA. Here, 19957 genes were initially screened and divided into 16 co-expressed modules (**Figure 4**). Among them, Darkgrey, Orange, Darkturquoise, Darklivegreen, Bisque4, and Plum1 were closely related to different growth and development stages, S1, S3, Z1, Z2, Z3, and Z5, respectively (**Figure 4**), suggesting that DEGs within modules may be involved in the growth, development, and secondary metabolic processes. Next, 9 modules related to terpenoid biosynthesis were identified, including Grey60, Lightcyan, Darkgrey, Orange, Darkmagenta, Plum1, Darkturquoise, Thistle2, and Salmon4, and a total of 28 key genes for terpenoid biosynthesis were screened in 9 modules (**Figure 4**; **Supplementary Table 6**). The core contribution of WGCNA is to cluster discrete DEGs into gene modules with collaborative expression patterns and identify the hub genes. Our findings provided a new perspective for understanding the regulatory network of terpenoid biosynthesis in *R. glutinosa* and screening the core nodes, which cannot be achieved by GO and KEGG analysis.

DEGs related to terpenoid skeleton biosynthesis, FPP/GGPP synthase, and iridoid glycoside biosynthesis were screened, in which the expression of terpenoid biosynthesis pathway genes was intuitively shown in the signaling pathway (**Figure 7**). To evaluate the roles of co-expression modules within genes, GO and KEGG enrichment analysis of key genes of 9 modules related to terpenoid biosynthesis was carried out. Based on GO analysis, key DEGs involved in terpenoid biosynthesis process were mainly enriched in secondary metabolism and secondary metabolite biosynthesis (**Figure 5**; **Supplementary Table 7**). KEGG analysis showed that key DEGs involved in terpenoid biosynthesis process were mainly enriched in sesquiterpene and triterpenoid biosynthesis, terpenoid skeleton biosynthesis, and terpenoid and polyketide metabolism (**Figure 6**). The expression levels, GO analysis, and KEGG enrichments suggested that these DEGs were related to terpenoid biosynthesis. Similarly, *RgFPPS* and *RgGGPS* were involved in the terpenoid biosynthesis in roots of *R. glutinosa* (Chen et al., 2021). *RgDXS*, *RgDXR*, *RgGPPS*, *RgG10H*, and *Rg10HGO* were key players in the terpenoids accumulation in *R. glutinosa* (Dong et al., 2022).

Further, the co-expression network of 28 hub genes and TFs (**Supplementary Table 8**) of 9 modules was constructed, respectively (**Figure 8**). The key genes involved in terpenoid biosynthesis through MVA pathway were mainly distributed in Darkagenta, Grey60, Lightcyan, Orange, and Plum1 (**Figures 8A, B, D–F**). *GRAS*, *GeBP*, *C3H*, *NF-YC*, *MYB-related*, *HD-ZIP*, *Trihelix*, *HSF*, *NAC*, *WRKY*, *bHLH*, or *ERF* may play a critical role in regulating *RgAACT1*~*3*, *RgMVK1*, *RgPMK1*, *RgPMK2*, or *RgHMGR1*~*3* expression. Research has shown that *bHLH*, *NAC*, and ethylene-sensitive transcription factors play important transcriptional regulatory roles in monoterpenes synthesis (Nieuwenhuizen et al., 2015; Van Moerkercke et al., 2015). The key genes related to MEP pathway in regulating terpenoid biosynthesis are mainly concentrated in Darkturquoise and Plum1 (**Figures 8C, F**). *EIL*, *WRKY*, *ERF*, or *bHLH* was involved in the regulation of expression of *RgDXS1*, *RgDXR1*, *RgMCT1*, *RgCMK1*, *RgHDS1*, or *RgMDS1*. In Darkturquoise, there is a direct synergistic regulation relationship among *RgDXS1*, *RgDXR1*, *RgMCT1*, *RgCMK1*, and *RgHDS1* (**Figure 8C**). Darkturquoise is specifically enriched in Z1 stage, indicating that the biosynthesis of terpenoid skeleton might be involved in Z1 stage. FPP/GGPP synthase appears in Plum1 module (**Figure 8F**), which is specifically enriched in Z5, indicating that the secondary metabolite accumulation may occur during Z5. *bHLH* and *ERF* play the crucial roles in regulating *RgGGPPS1*, *RgGGPPS2*, and *RgFPPS1* expression in Plum1. The key genes regulating iridoid glycosides biosynthesis were mainly concentrated in Plum1, Darkgrey, and Thistle2 (**Figures 8F–H**). *bHLH*, *ERF*, *MYB-related*, *HD-ZIP*, or *WRKY* was involved in the regulation of *Rg10HGO1*~*7* expression. In Changchun flowers, *bHLH* activates the key gene expression in the biosynthesis pathway of cyclohexene terpenes (Singh et al., 2021). These results were consistent with the research that *AP2/ERF*, *WRKY*, *MYB*, and *bHLH* could regulate the biosynthesis of pharmacological components (Hasunuma et al., 2008). So far, several large TFs, such as *MYB*, *WRKY*, *bHLH*, *ZIP*, *AP2/ERF*, have been predicted to regulate bioactive compound production, and these TFs positively or negatively regulate the expression of multiple hub genes, and thereby control the metabolic process of bioactive compounds (Wang et al., 2021b).

Among 28 genes related to terpenoid biosynthesis and metabolism pathways, 11 DEGs were not only co-expressed genes in various modules, but were key genes related to terpenoid biosynthesis. To evaluate the expression levels of 28 genes during 8 periods, their expression levels were shown by their FPKM values (**Figure 9A**), where 28 genes were highly expressed, respectively, at different stages. Subsequently, we validated the expression of 11 genes at different stages by RT-qPCR (**Figure 9B**), and the results were generally consistent with those in **Figure 9A**. Researchers have found that *DXS* and *DXR* overexpression have a regulatory effect on terpenoid biosynthesis in *Amomum villosum* (Yang et al., 2012). 11 key genes, except for *RgHMGR3*, were involved in MEP pathway and iridoid glycoside synthesis pathway. Our findings suggested that these key genes may be the rate-limiting factors for terpenoid accumulation. By analyzing previously reported metabolomic data of “Jinjiu” *R. glutinosa* at elongation stage (ER), expansion stage (TR), and maturity stage (MR) (respectively corresponding to S3, Z3, and Z5 in this study), we found that terpenoid biosynthesis-related precursors/intermediates were mainly enriched in ER or TR, and differential metabolites in TR or MR. Most of these terpenoids and their precursors/intermediates were annotated into secondary metabolite biosynthesis pathway (Zhou et al., 2018), consistent with the expression trends of terpenoid/terpenoid skeleton synthase-encoding genes (**Figure 9**). Moreover, integrated transcriptomic-metabolomic analyses confirmed *GES*, *CMK*, *DXS*, *HMGR*, *MVK*, *FPPS*, and/or *HDS* were the key genes in terpenoid and terpenoid skeleton biosynthetic pathways (Jiang et al., 2021; Xue et al., 2022; Maurya et al., 2023).

As a traditional Chinese medicine, the active components (especially terpenoids) of *R. glutinosa* played a crucial role in treating various diseases. Based on transcriptome data related to the growth and development of tuberous roots, the transcriptomic analysis was conducted to identify candidate genes potentially associated with terpenoid biosynthesis. However, the limitations of this study were lack of direct correlation analysis with metabolite content. Therefore, the following research will aim to verify the correlation between key genes and terpenoid accumulation through the joint analysis of metabonomic and transcriptomic data. Altogether, our findings provide the molecular basis for the study of terpenoid biosynthesis in *R. glutinosa* and a theoretical basis for improving the accumulation of terpenoid metabolites.

## Data Availability

The own experimental data that support the findings of this study are available from the corresponding author upon reasonable request. The RNA-Seq data of *Rehmannia glutinosa* underlying this paper were available in the NCBI SRA database (Accession numbers: PRJNA221109 and PRJNA197434).
