# Supplementary material for: Integrated transcriptomics and WGCNA reveal candidate hub genes associated with terpenoid biosynthesis in Rehmannia glutinosa
Source: Front Plant Sci. 2026 Jun 15;17:1834043. doi: 10.3389/fpls.2026.1834043 (PMC13311009; doi:10.3389/fpls.2026.1834043)
Supplement: Supplementary file 1 [file DataSheet1.docx]

**Supplemental Table S1 The specific primers of hub genes for qRT-PCR analysis**

| **Gene Name** | **Primer sequence (5'-3')** | **Gene ID** |
| --- | --- | --- |
| *RgHMGR3* | F: TTACCATGATGGAGGCTGTT | TRINITY_DN8138_c0_g1 |
|  | R: TTACCATGATGGAGGCTGTT |  |
| *RgMVK1* | F: ACCTCTGAGCTGCCATTAGG | TRINITY_DN10654_c0_g1 |
|  | R: GCTTCCCGTGGATTATCTTT |  |
| *RgFPPS1* | F: ATTCCCATAGCAATAAGCAC | TRINITY_DN11333_c0_g1 |
|  | R: GCATCAGGGCAGATGATAGA |  |
| *RgDXS1* | F: AACGCTGGTTTCCTCGATTC | TRINITY_DN10478_c0_g1 |
|  | R: TGTGCTTGTGGTCCGATTTG |  |
| *RgCMK1* | F: GCCCACAAAGCAGTAGCAGC | TRINITY_DN1851_c0_g1 |
|  | R: CGTTTATCAACCAACGTCCC |  |
| *RgMDS1* | F: TCAAAGAGGGAAACGAAA | TRINITY_DN11290_c0_g1 |
|  | R: TAAACCAGCGGACAGAAC |  |
| *RgHDS1* | F: AACAATGACTACAACCGATAC | TRINITY_DN11458_c0_g1 |
|  | R: GAATGTCCGCAACCAGA |  |
| *RgGES1* | F: TTGGGATGATCTTGGAAC | TRINITY_DN26481_c0_g1 |
|  | R: CTTTAATGATGGCTAATGG |  |
| *Rg10HGO1* | F: AATACGAACGACGAAATGCTC | TRINITY_DN12186_c0_g1 |
|  | R: GTGGTGACAGAGGTAGGGAAA |  |
| *Rg10HGO2* | F: GGGAGTATGATTGGAGGGCTAA | TRINITY_DN8497_c0_g1 |
|  | R: TCCCGACATCAATAACAAACCTAA |  |
| *Rg10HGO4* | F: AATGCGACCAATGCACTAAC | TRINITY_DN11314_c1_g1 |
|  | R: GTAATACCGGCACAAAGGAG |  |
| *RgGADPH* | F: TGCCTTGCTCCTTTGGCGAAGG |  |
|  | R: AAGCCGCTCTTCCACCTCTCCA |  |
| *RgTIP41* | F: TGGCTCAGAGTTGATGGAGTGCT |  |
|  | R: CTCTCCAGCAGCTTTCTCGGAGA |  |

**Supplemental Table S2 Quality statistics of transcriptome data from *R. glutinosa***

| Samples | Read number of raw data | Read number of clean data | GC Content | %≥Q30 |
| --- | --- | --- | --- | --- |
| S1 | 7,371,534 | 7,200,910 | 42.85 | 90.73 |
| S2 | 7,250,941 | 7,081,191 | 42.90 | 90.86 |
| S3 | 7,609,812 | 7,442,610 | 42.83 | 90.76 |
| Z1 | 6,297,907 | 6,283,585 | 44.66 | 96.32 |
| Z2 | 6,149,996 | 6,134,678 | 45.73 | 96.17 |
| Z3 | 6,216,743 | 6,203,125 | 44.54 | 96.32 |
| Z4 | 6,005,729 | 5,993,431 | 44.60 | 96.36 |
| Z5 | 6,195,513 | 6,175,052 | 47.33 | 95.66 |

**Supplemental Table S3 Statistical results of transcriptome alignment of *R. glutinosa***

| Samples | Comparison rate |
| --- | --- |
| S1 | 85.59% |
| S2 | 86.01% |
| S3 | 87.57% |
| Z1 | 85.86% |
| Z2 | 87.67% |
| Z3 | 85.77% |
| Z4 | 86.33% |
| Z5 | 88.77% |

**Supplemental Table S4 The key genes involved in terpenoids biosynthesis in *R. glutinosa***

| **Gene name** | **Number of Unigenes** |
| --- | --- |
| 1-Deoxy-D-xylulose 5-phosphate synthase (DXS) | 2 |
| CDP-ME kinase (CMK) | 1 |
| 2-C-methyl-D-erythritol 2,4-cyclodiphosphate synthase (MDS) | 1 |
| (E)-4-hydroxy-3-methylbut-2-enyl diphosphate synthase (HDS) | 1 |
| (E)-4-hydroxy-3-methylbut-2-enyl diphosphate reductase (HDR) | 1 |
| Geraniol synthase (GES) | 1 |
| 10-hydroxygeraniol oxidoreductase (10HGO) | 3 |
| HMG-CoA synthase (HMGS) | 1 |
| HMG-CoA reductase (HMGR) | 1 |
| Mevalonate kinase (MVK) | 1 |
| Phosphomevalonate kinase (PMK) | 1 |
| Mevalonate diphosphate decarboxylase (MVD) | 1 |
| Farnesyl diphosphate synthase (FPPS) | 1 |

**Supplemental Table S5 Gene ID of DEGs related to terpenoid biosynthesis in *R. glutinosa***

| **Gene Name** | **Gene ID** |
| --- | --- |
| *RgHMGS* | TRINITY_DN12303_c1_g1 |
| *RgHMGR* | TRINITY_DN8138_c0_g1 |
| *RgMVK* | TRINITY_DN10654_c0_g1 |
| *RgPMK* | TRINITY_DN2750_c0_g1 |
| *RgMVD* | TRINITY_DN10827_c0_g1 |
| *RgDXS* | TRINITY_DN17466_c0_g1 |
|  | TRINITY_DN10478_c0_g1 |
| *RgCMK* | TRINITY_DN1851_c0_g1 |
| *RgMDS* | TRINITY_DN11290_c0_g1 |
| *RgHDS* | TRINITY_DN11458_c0_g1 |
| *RgHDR* | TRINITY_DN12561_c0_g1 |
| *RgFPPS* | TRINITY_DN11333_c0_g1 |
| *RgGES* | TRINITY_DN21570_c0_g1 |
| *Rg10HGO* | TRINITY_DN12186_c0_g1 |
|  | TRINITY_DN11314_c1_g1 |
|  | TRINITY_DN8497_c0_g1 |

**Supplemental Table S6 Genes related to terpenoid biosynthesis in WGCNA**

| **Gene Name** | **Gene ID** | **Module** |
| --- | --- | --- |
| *RgAACT1* | TRINITY_DN10571_c0_g1 | grey60 |
| *RgAACT2* | TRINITY_DN11470_c1_g1 | lightcyan |
| *RgAACT3* | TRINITY_DN30050_c0_g1 | darkmagenta |
| *RgHMGR1* | TRINITY_DN22875_c0_g1 | grey60 |
| *RgHMGR2* | TRINITY_DN10027_c0_g1 | orange |
| *RgHMGR3* | TRINITY_DN8138_c0_g1 | plum1 |
| *RgHMGR4* | TRINITY_DN31066_c0_g1 | salmon4 |
| *RgMVK1* | TRINITY_DN10654_c0_g1 | darkmagenta |
| *RgPMK1* | TRINITY_DN17991_c0_g1 | darkmagenta |
| *RgPMK2* | TRINITY_DN37236_c0_g1 | darkmagenta |
| *RgFPPS1* | TRINITY_DN11333_c0_g1 | plum1 |
| *RgDXS1* | TRINITY_DN10478_c0_g1 | darkturquoise |
| *RgDXR1* | TRINITY_DN12531_c0_g1 | darkturquoise |
| *RgMCT1* | TRINITY_DN5756_c0_g1 | darkturquoise |
| *RgCMK1* | TRINITY_DN1851_c0_g1 | darkturquoise |
| *RgMDS1* | TRINITY_DN11290_c0_g1 | plum1 |
| *RgMDS2* | TRINITY_DN36174_c0_g1 | thistle2 |
| *RgHDS1* | TRINITY_DN11458_c0_g1 | darkturquoise |
| *RgGGPPS1* | TRINITY_DN2919_c0_g1 | plum1 |
| *RgGGPPS2* | TRINITY_DN17677_c0_g1 | plum1 |
| *RgGES1* | TRINITY_DN26481_c0_g1 | plum1 |
| *Rg10HGO1* | TRINITY_DN12186_c0_g1 | darkgrey |
| *Rg10HGO2* | TRINITY_DN8497_c0_g1 | darkgrey |
| *Rg10HGO3* | TRINITY_DN11657_c0_g1 | darkgrey |
| *Rg10HGO4* | TRINITY_DN11314_c1_g1 | plum1 |
| *Rg10HGO5* | TRINITY_DN11805_c0_g1 | plum1 |
| *Rg10HGO6* | TRINITY_DN11657_c2_g1 | plum1 |
| *Rg10HGO7* | TRINITY_DN28169_c0_g1 | thistle2 |

**Supplemental Table S7 GO enrichment analysis of co-expression modules related to terpenoid synthesis in *R. glutinosa***

| **Module** | **Gene number** | **GO_ID** | **Significantly enriched term** |
| --- | --- | --- | --- |
| grey60 | 9 | GO:0033645 | host cell endomembrane system |
|  | 9 | GO:0044165 | host cell endoplasmic reticulum |
|  | 9 | GO:0044167 | host cell endoplasmic reticulum membrane |
|  | 9 | GO:0033644 | host cell membrane |
|  | 9 | GO:0030430 | host cell cytoplasm |
| lightcyan | 6 | GO:0035371 | microtubule plus |
|  | 6 | GO:1990752 | microtubule end |
|  | 11 | GO:0006928 | movement of cell or subcellular component |
|  | 11 | GO:0030554 | nucleotide binding |
|  | 11 | GO:0003774 | nucleoside phosphate binding |
| darkgrey | 42 | GO:0019748 | secondary metabolic process |
|  | 32 | GO:1901657 | glycosylic compound metabolic process |
|  | 32 | GO:0044550 | secondary metabolite biosynthetic process |
|  | 27 | GO:0006928 | movement of cell or subcellular component |
|  | 23 | GO:0005875 | microtube associated complex |
| orange | 23 | GO:0010243 | response to organonitrogen compound |
|  | 20 | GO:0010200 | response to chitin |
|  | 14 | GO:0071229 | cellular response to acid chemical |
|  | 11 | GO:0071215 | cellular response to abscisic acid stimulus |
|  | 5 | GO:0035252 | UDP-xylosyltransferase activity |
| darkmagenta | 36 | GO:0006412 | transcription |
|  | 36 | GO:0043043 | peptide biosynthesis process |
|  | 32 | GO:0005198 | structural molecule activity |
|  | 28 | GO:0005840 | ribosome |
|  | 27 | GO:0044391 | ribosomal subunit |
| plum1 | 119 | GO:0005576 | extracellular region |
|  | 90 | GO:0048046 | apoplast |
|  | 85 | GO:0005198 | structural molecule activity |
|  | 80 | GO:0005840 | ribosome |
|  | 72 | GO:0003735 | structural component of ribosome |
| darkturquoise | 33 | GO:0019748 | secondary metabolic process |
|  | 27 | GO:0044550 | secondary metabolite biosynthetic process |
|  | 30 | GO:0071669 | plant-type cell wall organization or biogenesis |
|  | 18 | GO:0009698 | phenylpropanoid metabolic process |
|  | 16 | GO:0000502 | proteasome complex |
| thistle2 | 32 | GO:0009408 | response to heat |
|  | 28 | GO:0046686 | response to cadmium ion |
|  | 26 | GO:0006979 | response to oxidative stress |
|  | 20 | GO:0009636 | response to toxic substance |
|  | 18 | GO:0000302 | response to reactive oxygen species |
| salmon4 | 6 | GO:0042170 | plastid membrane |
|  | 5 | GO:0015934 | large ribosome subunit |
|  | 5 | GO:0044391 | ribosomal subunit |
|  | 5 | GO:0005840 | ribosome |

**Supplemental Table S8 Transcription factors regulating the terpenoids synthesis in 9 modules**

| Gene ID | Transcription factor family | Gene fnctional annotation | Module |
| --- | --- | --- | --- |
| TRINITY_DN5297_c0_g1 | C3H | C3H family protein | grey60 |
| TRINITY_DN10033_c0_g1 | HD-ZIP | Homeobox from Arabidopsis thaliana |  |
| TRINITY_DN7268_c0_g1 | Trihelix | Trihelix family protein |  |
| TRINITY_DN3682_c0_g1 | HSF | heat shock transcription factor A1D | lightcyan |
| TRINITY_DN25569_c0_g1 | NAC | NAC domain containing protein 58 |  |
| TRINITY_DN10033_c1_g1 | HD-ZIP | homeobox from Arabidopsis thaliana | darkgrey |
| TRINITY_DN32608_c0_g1 | MYB_related | MYB_related family protein |  |
| TRINITY_DN10442_c0_g1 | WRKY | WRKY DNA-binding protein 21 |  |
| TRINITY_DN12423_c0_g1 | GRAS | SCARECROW-like 14 | orange |
| TRINITY_DN9023_c0_g1 | WRKY | WRKY DNA-binding protein 33 |  |
| TRINITY_DN38248_c0_g1 | C3H | C3H family protein | darkmagenta |
| TRINITY_DN9301_c0_g1 | GeBP | DNA-binding protein-related transcriptional regulator |  |
| TRINITY_DN4835_c0_g1 | GRAS | scarecrow-like 3 |  |
| TRINITY_DN22238_c0_g1 | MYB_related | myb domain protein 73 |  |
| TRINITY_DN2207_c0_g1 | NF-YC | nuclear factor Y, subunit C13 |  |
| TRINITY_DN11373_c0_g1 | ARF | auxin response factor 4 | pluml |
| TRINITY_DN9017_c0_g1 | ARF | auxin response factor 6 |  |
| TRINITY_DN1240_c0_g1 | B3 | B3 family protein |  |
| TRINITY_DN10270_c0_g1 | BBR-BPC | basic pentacysteine 6 |  |
| TRINITY_DN26049_c0_g1 | BES1 | beta-amylase 2 |  |
| TRINITY_DN38065_c0_g1 | BES1 | BES1/BZR1 homolog 4 |  |
| TRINITY_DN10200_c0_g1 | bHLH | bHLH family protein |  |
| TRINITY_DN11017_c0_g1 | bHLH | bHLH family protein |  |
| TRINITY_DN11527_c0_g1 | bHLH | bHLH family protein |  |
| TRINITY_DN11570_c1_g2 | bHLH | bHLH family protein |  |
| TRINITY_DN26289_c0_g1 | bHLH | bHLH family protein |  |
| TRINITY_DN5688_c0_g1 | bHLH | bHLH family protein |  |
| TRINITY_DN764_c1_g1 | bHLH | bHLH family protein |  |
| TRINITY_DN9781_c0_g1 | bHLH | bHLH family protein |  |
| TRINITY_DN32782_c0_g1 | bZIP | ABA-responsive element binding protein 3 |  |
| TRINITY_DN3541_c0_g1 | bZIP | basic region/leucine zipper motif 27 |  |
| TRINITY_DN6771_c0_g1 | bZIP | basic region/leucine zipper transcription factor 16 |  |
| TRINITY_DN8346_c0_g1 | bZIP | basic leucine-zipper 7 |  |
| TRINITY_DN8380_c0_g1 | bZIP | basic region/leucine zipper motif 60 |  |
| TRINITY_DN36930_c0_g1 | C2H2 | C2H2 family protein |  |
| TRINITY_DN36937_c0_g1 | C2H2 | indeterminate（ID）-domain 2 |  |
| TRINITY_DN10644_c0_g1 | C3H | C3H family protein |  |
| TRINITY_DN11196_c0_g1 | C3H | CCCH-type zinc finger protein with ARM repeat domain |  |
| TRINITY_DN12322_c1_g1 | C3H | C3H family protein |  |
| TRINITY_DN12481_c0_g2 | C3H | C3H family protein |  |
| TRINITY_DN6429_c0_g1 | C3H | CCCH-type zinc finger protein with ARM repeat domain |  |
| TRINITY_DN9205_c0_g1 | C3H | floral homeotic protein （HUA1） |  |
| TRINITY_DN11940_c0_g1 | CAMTA | signal responsive 1 |  |
| TRINITY_DN12044_c0_g1 | Dof | TARGET OF MONOPTEROS 6 |  |
| TRINITY_DN11670_c0_g1 | ERF | ethylene responsive element binding factor 3 |  |
| TRINITY_DN12045_c0_g1 | ERF | related to AP2 4 |  |
| TRINITY_DN12278_c0_g1 | ERF | ethylene responsive element binding factor 4 |  |
| TRINITY_DN12536_c1_g1 | ERF | erf domain protein 9 |  |
| TRINITY_DN1267_c0_g1 | ERF | ERF family protein |  |
| TRINITY_DN18162_c0_g1 | ERF | ERF family protein |  |
| TRINITY_DN18176_c0_g1 | ERF | ERF family protein |  |
| TRINITY_DN9020_c0_g1 | ERF | ERF family protein |  |
| TRINITY_DN9402_c0_g1 | ARR | response regulator 2 |  |
| TRINITY_DN11701_c0_g1 | GATA | GATA transcription factor 11 |  |
| TRINITY_DN12055_c0_g1 | GATA | GATA transcription factor 9 |  |
| TRINITY_DN3798_c0_g1 | GATA | ZIM-like 1 |  |
| TRINITY_DN9595_c0_g1 | GeBP | DNA-binding protein-related transcriptional regulator |  |
| TRINITY_DN11146_c1_g1 | GRAS | GRAS family protein |  |
| TRINITY_DN12516_c0_g1 | GRAS | SCARECROW-like 8 |  |
| TRINITY_DN12625_c2_g1 | GRAS | GRAS family protein |  |
| TRINITY_DN14111_c0_g1 | GRAS | GRAS family protein |  |
| TRINITY_DN5525_c0_g1 | GRAS | GRAS family protein |  |
| TRINITY_DN6257_c0_g1 | GRAS | SCARECROW-like 21 |  |
| TRINITY_DN7061_c0_g1 | GRAS | GRAS family protein |  |
| TRINITY_DN9687_c0_g1 | GRAS | GRAS family protein |  |
| TRINITY_DN11626_c0_g1 | HD-ZIP | homeobox 1 |  |
| TRINITY_DN12405_c0_g1 | MYB | myb domain protein 73 |  |
| TRINITY_DN11076_c0_g1 | MYB_related | MYB_related family protein |  |
| TRINITY_DN36589_c0_g1 | NF-YA | nuclear factor Y, subunit A2 |  |
| TRINITY_DN10292_c0_g1 | NF-YB | nuclear factor Y, subunit B8 |  |
| TRINITY_DN25218_c0_g1 | NF-YB | nuclear factor Y, subunit B3 |  |
| TRINITY_DN7563_c0_g1 | NF-YC | nuclear factor Y, subunit C9 |  |
| TRINITY_DN1544_c0_g1 | SRS | Lateral root primordium （LRP） protein-related |  |
| TRINITY_DN23612_c0_g1 | TCP | TCP family protein |  |
| TRINITY_DN6783_c0_g1 | TCP | TCP family protein |  |
| TRINITY_DN10687_c0_g1 | Trihelix | Trihelix family protein |  |
| TRINITY_DN15447_c0_g1 | Trihelix | sequence-specific DNA binding transcription factors |  |
| TRINITY_DN5029_c0_g1 | Trihelix | Trihelix family protein |  |
| TRINITY_DN10608_c0_g1 | WRKY | WRKY DNA-binding protein 70 |  |
| TRINITY_DN22301_c0_g1 | AP2 | AP2 family protein | darkturquoise |
| TRINITY_DN24694_c0_g1 | AP2 | AP2 family protein |  |
| TRINITY_DN10073_c0_g1 | bHLH | bHLH family protein |  |
| TRINITY_DN12263_c0_g1 | EIL | EIL family protein |  |
| TRINITY_DN28515_c0_g1 | ERF | AP2 family protein |  |
| TRINITY_DN28452_c0_g1 | G2-like | G2-like family protein |  |
| TRINITY_DN27141_c0_g1 | MYB | myb domain protein 112 |  |
| TRINITY_DN32261_c0_g1 | NAC | NAC domain containing protein 61 |  |
| TRINITY_DN4067_c0_g1 | NAC | NAC domain containing protein 90 |  |
| TRINITY_DN6184_c0_g1 | WRKY | WRKY DNA-binding protein 28 |  |
| TRINITY_DN1650_c0_g1 | EIL | EIL family protein | thistle2 |
| TRINITY_DN7597_c0_g1 | ERF | related to AP2 4 |  |
| TRINITY_DN10722_c1_g2 | MYB_related | myb domain protein 70 |  |
| TRINITY_DN24174_c0_g1 | MYB_related | myb domain protein r1 |  |
| TRINITY_DN27821_c0_g1 | Trihelix | Trihelix family protein |  |

**
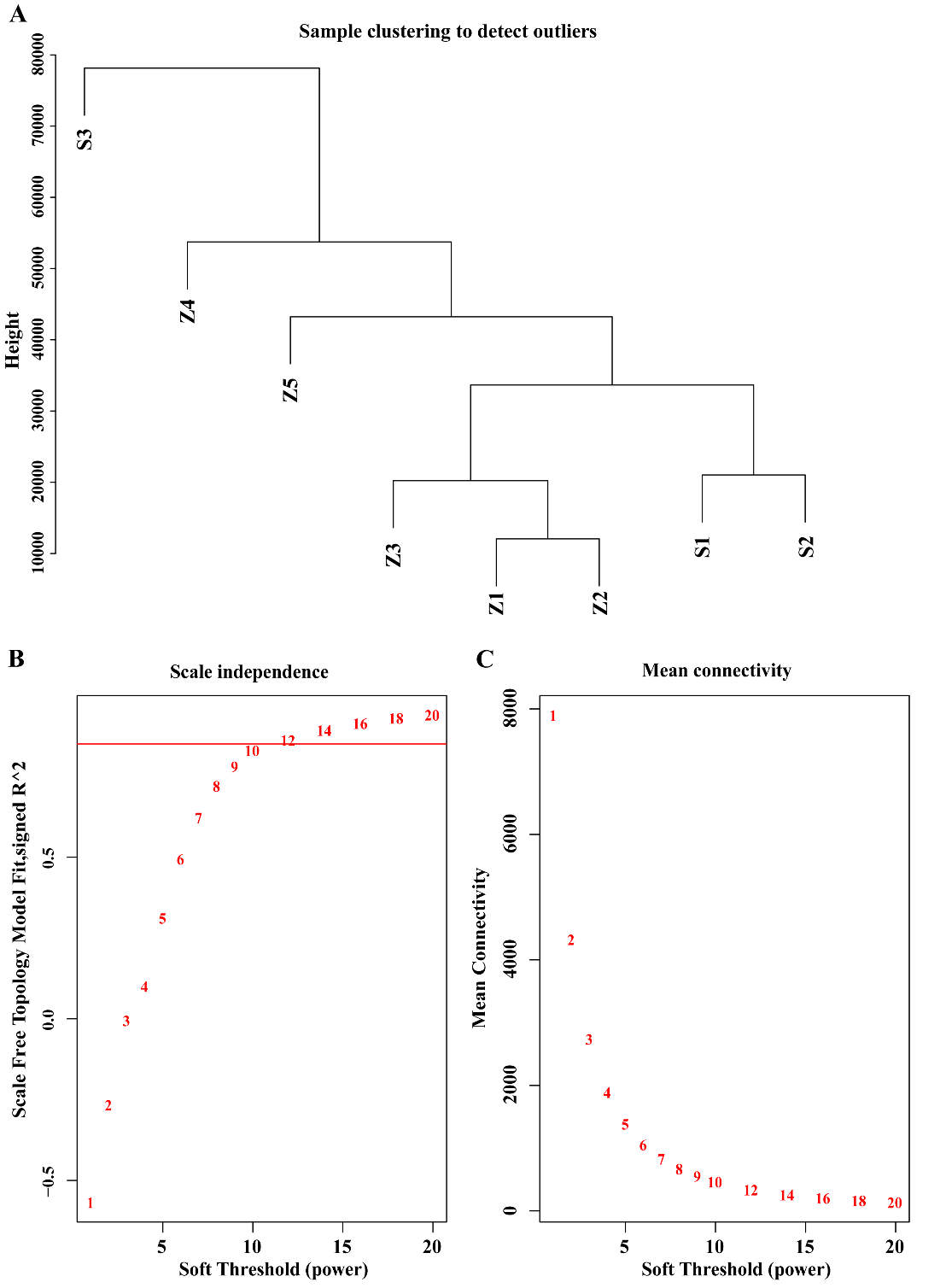
Supplemental Figure S1**

**Supplemental Figure S1 Sample clustering and the determination of soft threshold.**  **(A)** Cluster topology diagram of 8 groups of samples. **(B-C)** The horizontal axis represents the soft threshold β. The vertical axis of the left figure **(B)** represents the square of the correlation coefficient in the corresponding network. The vertical axis of the right figure **(C)** represents the mean of all gene adjacency functions in the corresponding gene module. The approximate scale-free topology can be attained at the soft thresholding power of 12.
